# Spatial Network Mapping of Pulmonary Multidrug-Resistant Tuberculosis Cavities Using RNA Sequencing

**DOI:** 10.1164/rccm.201807-1361OC

**Published:** 2019-08-01

**Authors:** Keertan Dheda, Laura Lenders, Shashikant Srivastava, Gesham Magombedze, Helen Wainwright, Prithvi Raj, Stephen J. Bush, Gabriele Pollara, Rachelle Steyn, Malika Davids, Anil Pooran, Timothy Pennel, Anthony Linegar, Ruth McNerney, Loven Moodley, Jotam G. Pasipanodya, Carolin T. Turner, Mahdad Noursadeghi, Robin M. Warren, Edward Wakeland, Tawanda Gumbo

**Affiliations:** ^1^ Centre for Lung Infection and Immunity, Division of Pulmonology, Department of Medicine and UCT Lung Institute and South African MRC/UCT Centre for the Study of Antimicrobial Resistance, University of Cape Town, Cape Town, South Africa; ^2^ Faculty of Infectious and Tropical Diseases, Department of Immunology and Infection, London School of Hygiene and Tropical Medicine, London, United Kingdom; ^5^ Department of Pathology; ^7^ Department of Nuclear Medicine, and; ^8^ Chris Barnard Division of Cardiothoracic Surgery, Department of Surgery, Groote Schuur Hospital, University of Cape Town, Cape Town, South Africa; ^3^ Center for Infectious Diseases Research and Experimental Therapeutics, Baylor Research Institute, Baylor University Medical Center, Dallas, Texas; ^4^ Department of Immunology, University of Texas Southwestern Medical Center, Dallas, Texas; ^6^ Division of Infection and Immunity, University College London, London, United Kingdom; and; ^9^ South African Medical Research Council Centre for Tuberculosis Research/Department of Science and Technology/National Research Foundation Centre of Excellence for Biomedical Tuberculosis Research, Division of Molecular Biology and Human Genetics, Stellenbosch University, Cape Town, South Africa

**Keywords:** transcriptomics, pulmonary tuberculosis, *in silico* analysis, TB cavitation

## Abstract

**Rationale:** There is poor understanding about protective immunity and the pathogenesis of cavitation in patients with tuberculosis.

**Objectives:** To map pathophysiological pathways at anatomically distinct positions within the human tuberculosis cavity.

**Methods:** Biopsies were obtained from eight predetermined locations within lung cavities of patients with multidrug-resistant tuberculosis undergoing therapeutic surgical resection (*n* = 14) and healthy lung tissue from control subjects without tuberculosis (*n* = 10). RNA sequencing, immunohistochemistry, and bacterial load determination were performed at each cavity position. Differentially expressed genes were normalized to control subjects without tuberculosis, and ontologically mapped to identify a spatially compartmentalized pathophysiological map of the cavity. *In silico* perturbation using a novel distance-dependent dynamical sink model was used to investigate interactions between immune networks and bacterial burden, and to integrate these identified pathways.

**Measurements and Main Results:** The median (range) lung cavity volume on positron emission tomography/computed tomography scans was 50 cm^3^ (15–389 cm^3^). RNA sequence reads (31% splice variants) mapped to 19,049 annotated human genes. Multiple proinflammatory pathways were upregulated in the cavity wall, whereas a downregulation “sink” in the central caseum–fluid interface characterized 53% of pathways including neuroendocrine signaling, calcium signaling, triggering receptor expressed on myeloid cells-1, reactive oxygen and nitrogen species production, retinoic acid–mediated apoptosis, and RIG-I-like receptor signaling. The mathematical model demonstrated that neuroendocrine, protein kinase C-θ, and triggering receptor expressed on myeloid cells-1 pathways, and macrophage and neutrophil numbers, had the highest correlation with bacterial burden (*r* > 0.6), whereas T-helper effector systems did not.

**Conclusions:** These data provide novel insights into host immunity to *Mycobacterium tuberculosis*–related cavitation. The pathways defined may serve as useful targets for the design of host-directed therapies, and transmission prevention interventions.

**At a Glance Commentary**

**Scientific Knowledge on the Subject**

Host immunity to *Mycobacterium tuberculosis* and the pathogenesis of cavitation, the key propagation mechanism of tuberculosis (TB), are poorly understood. There are hardly any data about the TB cavity because most studies have focused on the granuloma.

**What This Study Adds to the Field**

To our knowledge, this is the first study to interrogate the host transcriptome and characterize pathophysiological mechanisms at anatomically distinct locations within TB cavities. TB cavities were characterized by a centralized “sink” with profound downregulation of numerous immune pathways toward the cavity center, including a newly described neuroendocrine pathway, which correlated with poor bacterial containment.

Tuberculosis (TB), first described in the *Rigveda* in India 3,500 years ago, has killed more than 1 billion people over the past two centuries, and remains the commonest infectious cause of death in many countries (1). Moreover, the advent of multidrug-resistant (MDR) TB threatens to wipe out recent gains in TB control (2, 3). An effective vaccine and/or immunotherapeutic intervention combined with interruption of transmission offer the only tangible hope of eliminating the disease. However, several recent TB vaccine candidates have failed to show clinical efficacy, or at best were only partially effective, despite promising data from animal studies (4–7). One reason may be lack of detailed knowledge about protective host immunity and the pathophysiology of cavitation. Previous work on granulomas emphasized the role of T-helper cell type 1 (Th1) immunity, CD4 T cells, IFN-γ signaling, IL-12, tumor necrosis factor (TNF)-α, eicosanoid signaling, and lipid dysregulation (8–12). One recent proteomics study found that the granuloma center was dominated by proinflammatory responses, whereas the area outside had an antiinflammatory signature (12). It is unclear if the same pathways drive cavitation or *Mycobacterium tuberculosis* (*Mtb*) replication in TB cavities. Cavitation underpins liquefactive necrosis, bacterial aerosolization, and hence disease transmission.

Although often arising from it, the pulmonary cavity is not the same as the granuloma. Over the last four centuries, the histology of TB cavities has been described based on autopsy work from Europe and the United States (13–15). Recent histological MDR-TB case reports from South Africa suggest a picture of failed immunity at the luminal edge of the TB lung cavity (16). The cause is unclear, but likely *Mtb* itself could have an immunosuppressive role via either release of mediators, as we have explored elsewhere, or by directly killing immune cells (17). But why, how, and exactly where does host immunity fail to control *Mtb* growth in this lesion? To answer these questions, we conducted a case-control clinical study where we performed RNA sequencing (RNA-Seq) on biopsies from anatomically distinct points within lung cavities of patients with MDR-TB and compared them with healthy lung tissue from those without TB (control subjects). Data were analyzed using standard modular and unsupervised approaches and subsequently modeled using our recently derived dynamical sink model (18).

## Methods

### Patient Recruitment and Dissection Procedures

Between 2012 and 2013, we recruited patients referred to Groote Schuur hospital for therapeutic surgical resection after failed MDR-TB chemotherapy (18). Control subjects were patients undergoing lung surgery for non-TB reasons, with no clinical or radiological features of TB. After surgery, the resected lung was immediately placed on ice, and transported to the BSL3 laboratory (∼200 m from the operating room).

Surgical dissection procedures were performed to avoid cross-contamination between biopsy positions, as previously described (18). Multiple 2-mm biopsies were taken at seven anatomically distinct sites within the cavity: *1*) normal-appearing lung tissue 2–5 cm from the fibrotic cavity edge, *2*) perifibrotic cavity edge, *3*) center of the cavity wall, *4*) luminal edge of the cavity wall, *5*) air–caseum interface at cavity lumen center, *6*) airways greater than or equal to 2.5 cm distal, and *7*) proximal to cavity mouth; and *8*) sputum. In control subjects, similarly sized biopsies were obtained from a single lung position. Each biopsy, including sputum, was *1*) immediately placed in RNAlater for RNA-seq, *2*) cultured for *Mtb* using mycobacterial growth indicator tube system, and *3*) fixed in formalin for histopathology (hematoxylin and eosin staining) and immunohistochemistry.

RNA extraction, sequencing and quality control were performed accordingly. Mycobacterial growth indicator tubes were monitored for time-to-positivity, and expressed as *Mtb* cfu/g of tissue. Immunohistochemistry was performed to corroborate RNA-Seq analysis findings. Full methodology is provided in the online supplement.

### Dual Positron Emission Tomography/Computed Tomography Scanning and Reading

Positron emission tomography/computed tomography (PET-CT) imaging was performed in accordance with the globally accepted fluorodeoxyglucose PET/CT EANM procedure guidelines for tumor imaging (19). Measurements of cavity volume are detailed in the online supplement.

### Statistical and Bioinformatics Analyses

Differentially expressed genes (DEGs), compared with non-TB control tissue, were defined as greater than log_2_ change and a Benjamini-Hochberg adjusted *P* less than 0.01. Modular analyses were performed using prevalidated and annotation-specific modules, as previously described (20–24). Reads were expressed per kilobase per million mapped reads (RPKM) for constituent genes of each module. Established immune pathways associated with DEGs were identified using ingenuity pathway analysis (IPA). We interrogated the whole transcriptome in an agnostic and unbiased fashion by identifying the most extensively changed physiological processes in the TB cavity.

### Mathematical Model

RNA-Seq data were modeled to explore the relationship with bacterial burden and to integrate immune pathway interactions. Given that PET-CT scans and histological and RNA-Seq analyses revealed pathophysiological patterns consistent with our recently derived dynamical “sink” model (18), we modified standard linear models to incorporate these dynamical “sinks.” In a dynamical system, a point or state (e.g., cavity position) evolves over time according to specified rules (25). We modified the state to evolve over distance in the cavity rather than time. This model was then used to map the interaction of different pathways along the cavity positions, as detailed in the online supplement.

## Results

### Clinical and Radiological Characteristics of Patients and Control Subjects

Clinical features describing the 14 patients with MDR-TB (11 MDR plus resistance to other drugs) are shown in Table E1 in the online supplement. The median (range) age was 33 (14–50) years. Two patients were HIV coinfected, but were on effective antiretroviral therapy. Typical preoperative PET-CT scans showed lesions consisting of consolidation with central cavitation and a median (range) lung cavity volume of 50 cm^3^ (15–389 cm^3^) (*see* Figure E1 and Table E1). Among the 10 control patients without TB, the median age was 30 (23–74) years. One control subject had HIV-infection and was on antiretroviral therapy.

### Pathological and Microbiological Characteristics

Gross pathological examination of resected TB-infected lungs revealed a median of two (one to three) cavities per resected specimen, with a diameter of 4 cm (2–8 cm) per cavity. Hematoxylin and eosin stains revealed site-specific histology (*see* Figure E2 and Table E1). The cavity wall was characterized by fibrosis and chronic inflammation (20 ± 7.7% and 35 ± 11% of the biopsied area, respectively; *P* = 0.014). Cell populations in the cavity wall included 14% (4.2–40%) neutrophils and 20% (8.9–40%) histiocytes (*P* = 0.34). *Mtb* could be visualized at all cavity positions, including normal-appearing tissue, both extracellularly and within neutrophils or macrophages. Lung tissue in control patients demonstrated normal histology. *Mtb* was culturable at all cavity positions, including normal-appearing lung tissue. However, the highest bacterial burden (i.e., lowest time-to-positivity) was at the air–caseum interface. No *Mtb* growth occurred in lung tissue from control patients (*see* Figure E3).

### RNA Sequencing Results and Principal Component Analyses

Sixty-nine samples passed the stringent RNA-Seq quality criteria detailed in the online supplement. Reads were aligned to human genome, and were mapped to the 19,049 genes; 31.07% were splice variants (*see* Figure E4). All RNA-Seq reads were examined *in toto* using principal component analysis (*see* Figure E5). Transcripts clustered into four main groups: control subjects (i.e., no TB), normal-appearing tissue, the combination of three cavity wall positions, and a distinct subcluster of cavity center samples within the normal-appearing tissue cluster. Therefore, we assigned all samples to one of six groups for further analyses: *1*) control subjects (non-TB), *2*) normal-appearing tissue, *3*) cavity wall, *4*) air–caseum interface, *5*) sputum, and *6*) airways.

### Modular Analyses to Map Cell Types and Effectors to Cavity Positions

**Figure 1.**
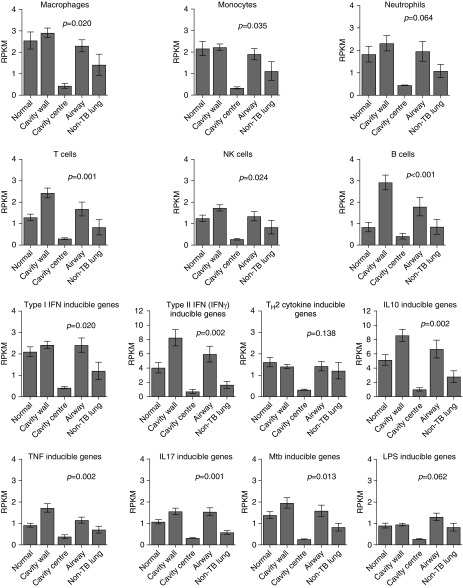
Cell type and effector responses distributions in and around cavity. The graphs show the mean ± SEM of per kilobase per million mapped reads (RPKM) of all genes in the module at each position; “Normal” is normal-appearing lung in tuberculosis (TB), whereas “Non-TB lung” refers to the control subjects. Cavity wall positions were combined. The Kruskal-Wallis test *P* value (corrected for multiple comparisons) in each graph is shown for the comparison of RPKM values in control subjects without TB versus RPKM values in the different cavity positions. For RPKM comparison between control subjects without TB and normal-appearing lung tissue in patients with multidrug-resistant TB, *P* value was >0.2 for all comparisons. The RPKM values were higher in macroscopically normal-appearing tissue in patients with multidrug-resistant TB compared with non-TB control lung, but were highest in the cavity wall, with a precipitous decline below non-TB lung in the cavity center. However, expression of Th2 cytokine–inducible genes and LPS response were not higher in patients with multidrug-resistant TB than in control subjects. The highest RPKM values relative to control subjects were with the type II IFN (IFN-γ)–inducible genes in the cavity wall. Also notable are the increases in B cells in the cavity wall and airways. Mtb = *Mycobacterium tuberculosis*; NK = natural killer; Th2 = T-helper cell type 2; TNF = tumor necrosis factor.

We used our validated modular analysis approach on the RNA-seq data to map modules to cavity positions (20–24). Figure E6 shows multiple upregulated and downregulated genes within each module, indicating that modules were not influenced by a single highly expressed gene. Figure 1 shows modules for six cell types. In most cases cells were most abundant in the cavity wall compared with control subjects, with the notable exception the module for natural killer cells. Conversely, the air–caseum interface at cavity center had the lowest cellular abundance. A similar pattern was observed in modules of effector functions, with the exception of Th2 and LPS responses (Figure 1). Overall, the modular analysis indicates that each cavity position had a remarkably consistent gene expression profile between different patients, with small error bars between patients, and was independent of cavity size and disease extent.

### Unsupervised Analyses Using the Highest Differentially Expressed Pathways

**Figure 2.**
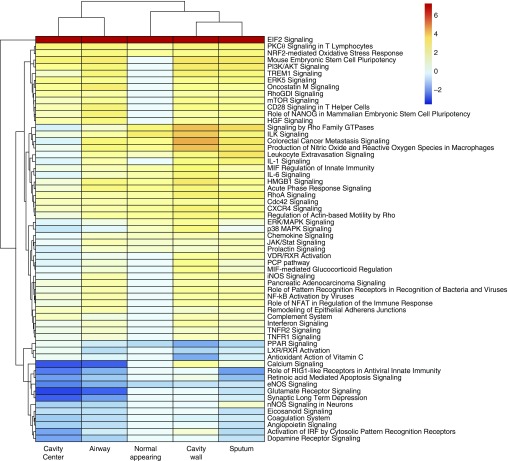
A cavity map of the 60 highest differentially expressed pathways. The fold change scale for the pathways is shown using a log_2_ scale (e.g., 6 on this scale is 64-fold change). The fold change is relative to nontuberculosis lung from control subjects, not normal-appearing tissue from subjects with tuberculosis. There is a functional “hole” in the cavity center, as can be seen by amount of pathways with blue color. However, not all pathways were downregulated in the cavity center; it can be seen that up to a quarter were actually upregulated. In contradistinction, the cavity wall had the most pathways upregulated and had the most intensely upregulated pathways of all. There are several prominent “nonimmunological” pathways that were among the top 60 expressed pathways, including, for example, colorectal cancer metastasis signaling via WNT signaling, and p38 MAPK signaling, downregulated in the cavity center but upregulated in the cavity wall.

DEGs were mapped to different physiological pathways using IPA. To avoid bias when choosing the important pathways, we defined “important” as the highest differentially expressed pathways (up or down), regardless of whether they had known immunological function or not. IPA of the top 500 DEGs identified 60 physiological pathways that are shown in Figure 2. Different expressed pathways were confined to specific spatial locations within each cavity. Importantly, IPA results were consistent with our modular analyses findings in Figure 1, indicating that we reached the same conclusions using two different analytic approaches. These data in Figure 2 illustrate that the topology-constrained organization of interlinked pathways remained consistent regardless of cavity size and volume (which showed significant variation between patients). Thus, the TB cavity is mathematically an “attractor,” defined as the condition toward which trajectories of systems converge despite different starting conditions, in nonlinear dynamical systems.

### Complex Pathway Expression Is Constrained by Cavity Topology

**Figure 3.**
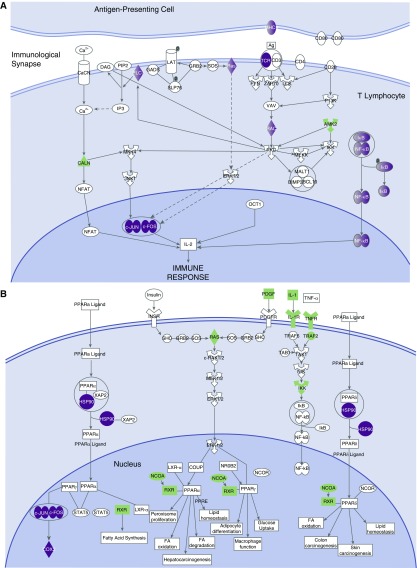
Highly expressed pathways in normal-appearing tissue. Purple denotes proteins encoded by upregulated genes, and green denotes downregulated genes. (*A*) Serine/threonine-specific protein kinase C-θ signaling. Shown are upregulated MHCII, TCR, and downstream RAC genes; also shown is upregulation of the linked NFκB and c-JUN/c-FOS transcription signaling pathways. This suggests ongoing antigen presentation and processing and ongoing antiapoptotic signaling. (*B*) PPAR-α (peroxisome proliferator–activated receptor-α) signaling genes are shown downregulated in normal-appearing tissue. The consequences of the downregulation of PPAR-α are unclear; however, it is a selective negative regulator of T-helper cell type 17 differentiation and positive regulator for regulatory T cells. Thus, consequences could be increased T-helper cell type 17 expression and decreased regulatory T-cell expression.

In Figure 2, normal-appearing lung tissue had the least number of significantly regulated pathways of any spatial position (14/60 [23%]). Of note, serine/threonine-specific protein kinase C-θ (PKCθ) signaling was observed to be upregulated in normal-appearing lung tissue, suggesting ongoing antigen presentation at the immunological synapse and thus T-cell proliferation (Figure 3A). However, peroxisome proliferator–activated receptor-α signaling, and the related liver X receptor and retinoid acid receptor signaling, were the most downregulated, as shown in Figure 3B.

In contrast, 47/60 (78%) of pathways in the cavity wall demonstrated increased expression in Figure 2. Only peroxisome proliferator–activated receptor-α and liver X receptor and retinoid acid receptor, vitamin C antioxidant activation, and endothelial nitric oxide synthase signaling were downregulated in the cavity wall. The cavity wall had the highest expression of all pathways, including nitric oxide production and reactive oxygen species by macrophages, IL-1, IL-6, IFN-γ, and nuclear factor-κB (NF-κB) activation. Of note, triggering receptor expressed on myeloid cells-1 (TREM-1) signaling was one of the most highly expressed pathways. In addition, 15/60 (25%) highly expressed pathways changed in parallel with TREM-1 across cavity positions. Many of these, such as PI3K/AKT, ERK/MAPK, p38 MAPK, IL-1, IL-6, chemokine, NF-κB, IFN-γ, TNFR1, TNFR2, IL-8, IL-10, monocyte chemotactic protein 1, and cell adhesion via CD54, are downstream to, and are upregulated by, TREM-1 signaling (25–28). Thus, TREM-1 and the 15 related signaling pathways formed dynamic networks of interactions that changed in parallel at this cavity position, and are by definition a complex system.

**Figure 4.**
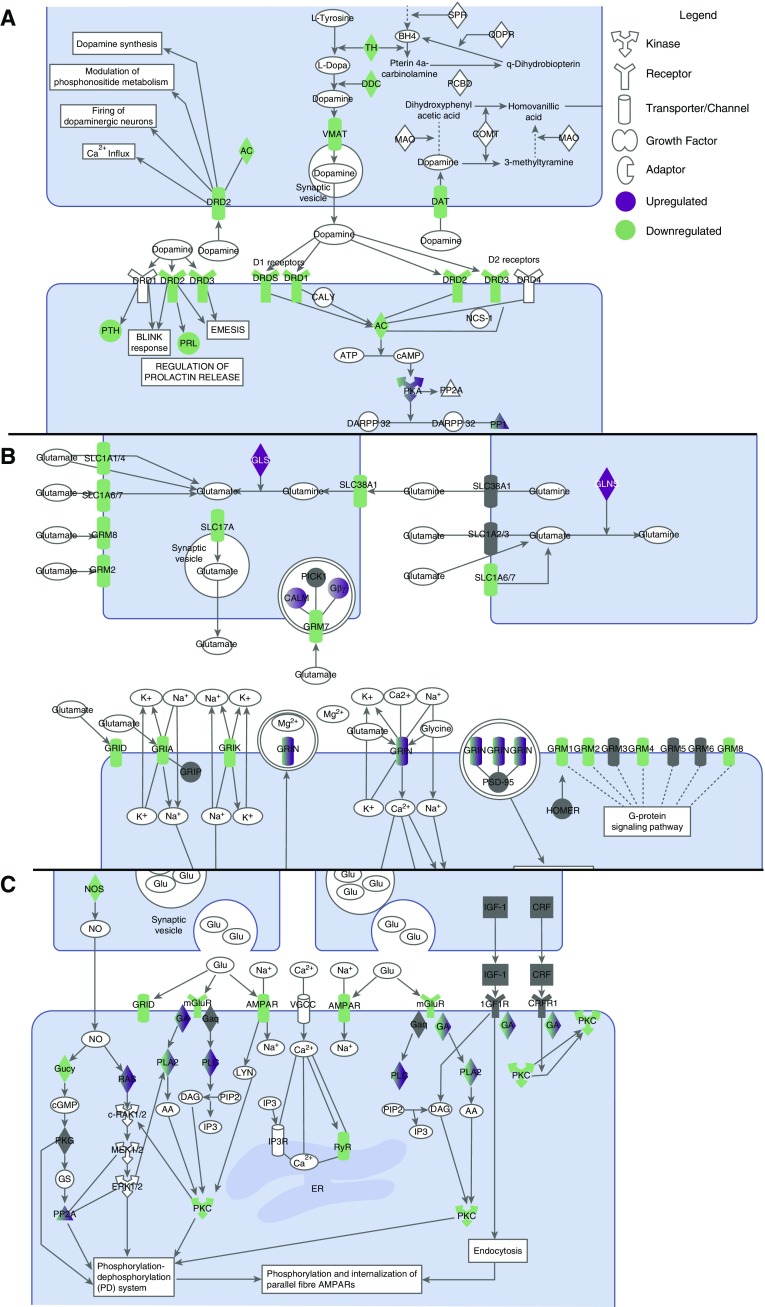
Neuroendocrine signaling pathways in the cavity center. (*A*) Dopamine receptor signaling in the cavity center showed decreased expression of genes encoding three of the five dopamine and uptake receptors, and the downstream parathyroid hormone and prolactin pathways. (*B*) Glutamate receptor signaling showed decrease in the expression of the two varieties of receptors: ionotropic (iGluR) and metabotropic receptors. Genes for each iGluR pharmacological class were downregulated: group I genes (*GRM1* and *GRM5*) activate phospholipase C, and both group II (*GRM2*) and group III (*GRM4*, *GRM7*, and *GRM8*) genes inhibit the cyclic AMP cascade. (*C*) In synaptic long-term depression, α-amino-3-hydroxy-5-methyl-4-isoxazolepropionic acid receptor (gene *AMPAR*), which works via *PLA2* (phospholipase A2) and *PKC* (protein kinase C), was downregulated, as were *PLA2* and *PKC*. iGluR AMPAR genes, such as *GRIA* and *GRIN*, were also downregulated, as were genes encoding the vesicular glutamate transporters SLC17A and SLC1A6/7. The related calcium signaling is shown in Figure E7. GluR = glutamate receptor; mGluR = metabotropic glutamate receptor; PRL = prolactin; PTH = parathyroid hormone.

The air–caseum interface (cavity center) exhibited the highest number (32/60 [53%]) of downregulated pathways, including RIG-I–like receptors, retinoic acid-mediated apoptosis, and hitherto described TREM-1–linked pathways. The most distinct feature of Figure 2 was that 5/32 (16%) of the downregulated pathways at the air–caseum interface were neuroendocrine-related (dopamine-, metabotropic glutamate receptor (mGluR)-, mGluR-dependent synaptic long-term depression formation; neuronal nitric oxide synthase, and prolactin-signaling), several of which are shown in Figure 4. Altogether there were more than 30 downregulated DEGs mapping to this neuroendocrine system, which were the most intensively downregulated genes in the entire transcriptome. Closely related was the decrease in Ca^2+^ signaling, including of receptor-operated Ca^2+^ channels that are activated by binding of neurotransmitters (*see* Figure E7). We propose these pathways as constituting the neuroendocrine system. In contrast, complement, which has been linked to control of *Mtb* burden, was one of the few pathways upregulated at the air–caseum interface (29, 30).

In the airways, 18/60 (30%) pathways were extensively downregulated, whereas 36/60 (60%) were upregulated (Figure 2). Thus, the airways had mixed picture that only partially reflected the rest of the TB cavity.

### Ontological Integration of Multiple Networks and *Mtb* Burden by Spatial Location

**Figure 5.**
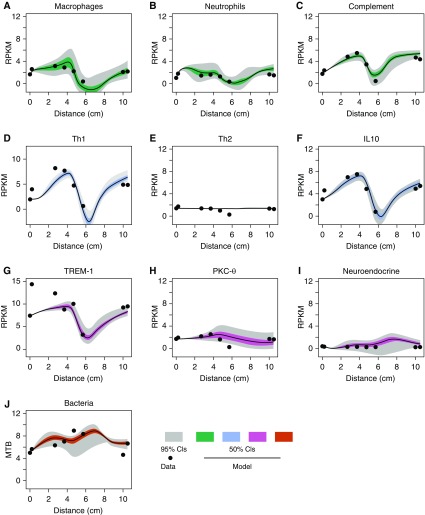
Model fit for dynamical sink with coupled interaction network. Model fits are shown with mean per kilobase per million mapped read (RPKM) values for each module (consisting of all genes in the module), for (*A*) macrophages, (*B*) neutrophils, (*C*) complement, (*D*) Th1, (*E*) Th2, (*F*) IL-10, (*G*) TREM-1, (*H*) protein kinase C-θ, (*I*) neuroendocrine, and (*J*) *Mycobacterium tuberculosis* burden. There were deep wells for the sink encountered for macrophages (*A*), complement (*C*), Th1 (*D*), IL-10 (*F*), and TREM-1 (*G*) at 6–8 cm. The neuroendocrine system/pathway looks flat because of the scale used and the relatively low RPKM reads (mostly downregulated compared with nontubersulosis lung). The scaled-up figure is shown in Figure E8. Table E3 shows the dynamical sink parameters of the neuroendocrine module. CI = confidence interval; Mtb = *Mycobacterium tuberculosis*; PKCθ = protein kinase C-θ; Th1 = T-helper cell type 1; TREM-1 = triggering receptor expressed on myeloid cells-1.

Given the multiplicity (60) of pathways, we sought to limit complexity and to organize the information using mathematical modeling. Our nonlinear dynamical sink model mapped along the TB cavity space resulted in an excellent fit for the multiple pathways and the associated *Mtb* burden as shown in Figure 5 and Figure E8, whereas standard linear models did not (*see* Figures E9 and E10) (*see* online supplement for a detailed explanation) (18, 25). The model parameter estimates for the dynamical sink model are shown in Table E2. Thus, the multiple pathways formed complex systems that had nonlinear and spatially constrained interactions, which fulfills the definition of complex adaptive systems (31–33).

Next, we calculated partial rank correlation coefficients between both observed and Latin hypercube sampling–based RPKM values of the different effector modules, with results shown in Figure E11. Latin hypercube sampling magnified the resolution of the observed RPKM values and allowed us to examine dynamical sink model sensitivity. Figures E11A and E11B shows strong correlation (i.e., *r* > 0.6 or < −0.6) between macrophage and neutrophil counts and both complement stimulation and expansion, but negative correlation with macrophage and neutrophil infection and bursting at different cavity positions. Infected macrophages and neutrophils burst after intracellular bacteria replication and are assumed to release more than 25 *Mtb* per cell (termed “burst size”) into the extracellular environment (hence increasing extracellular bacterial burden and infecting more cells), as part of necrosis, based on prior studies (34–36). The assumption of necrosis as opposed to apoptosis was based on the finding that apoptosis signaling was not upregulated in cavities; indeed, retinoic acid–mediated apoptosis was significantly downregulated. Extracellular *Mtb* burden varied by cavity position (*see* Figure E3 and Table E1), which differentially influenced immune cell abundance by cavity position. The pattern of cell abundance could be explained either via influx of macrophages and neutrophils to the respective locations, or increased expression, or differential location dependent immune cell death rates (which were all captured in the model): the results of the modeling suggest that depletion of different immune cells, such as macrophages and neutrophils, and other cell types in the “sink” is caused by *Mtb* killing of the immune cells.

### Neuroendocrine and PKCθ Expression Correlation with *Mtb* Burden

The mathematical model allowed us to explore the correlation between *Mtb* burden and different physiological pathways, based on *in silico* simulations plus observed data (*see* Figure E12). There was poor correlation between bacterial burden and either Th1 or Th2 system expression, whereas TREM-1 expression showed good correlation only in the cavity wall (*see* Figures E12A–E12F). Uniquely, PKCθ demonstrated negative correlation with *Mtb* burden that was highest in the cavity wall (*see* Figure E12G). Neuroendocrine expression had high negative correlation with bacterial burden: bacterial burden increased with increased neuroendocrine expression (*see* Figure E12H).

### Model Simulation-Perturbation Experiments

What happens to *Mtb* burden and each physiological module at each cavity position when a particular pathway is either stimulated or inhibited to a specified degree *in silico*? An example is shown for the neuroendocrine system in Figure E13. The simulations predict profound neuroendocrine system “dose-dependent” changes in bacterial burden in Figure E13A, with increase in *Mtb* burden as neuroendocrine signaling increases. Figures E13B–E13I show negative relationships between neuroendocrine expression and expression of Th1, IL-10, complement, and infection of macrophages and neutrophils. These relationships were constrained by spatial position.

### Immunohistochemistry Confirmation of RNA-Seq and Model Findings

We performed immunohistochemistry on the cavity wall, airways, and control lung tissue, to confirm the RNA-Seq and mathematical modeling findings. Figure E14 shows that the abundance of CD4^+^ and CD8^+^ was similar between the cavity wall, airways, and noninfected control subjects. For the macrophage lineage, Figure E14C demonstrated a 1.49-fold higher CD68^+^ cell population (i.e., macrophage lineage) in the cavity wall compared with noninfected control subjects, with similar levels in the airways. FOXP3^+^ cell stains revealed a 15-fold higher abundance of FOXP3^+^ cells (regulatory T cells) in the cavity wall compared with airways (*see* Figure E14D). For the proposed neuroendocrine system, we stained for chromogranin A (parathyroid hormone secretory system specific) in airways, the cavity wall, and in control subjects, with results shown in Figure E14E. The airways had lower chromogranin ratios than the cavity wall, and were lower than in control subjects without TB, consistent with RNA-seq findings and our dynamical sink model. We also used a neuroglia-specific S100B stain, and the airways showed significantly lower intensity for S100B stain than the cavity wall (*see* Figure E14F). For another confirmation, cavity wall biopsy neurofibrin staining was positive in four of eight patient cavities tested.

## Discussion

Our main goal was to identify how and where host immunity failed to control *Mtb* burden in MDR-TB cavities, and to identify the specific host immunity pathways that failed. We identified at least 60 different highly expressed pathways that formed complex adaptive system networks, which we integrated using a dynamical sink model onto approximately 600 cm^3^ histopathological space/tissue volume. In the cavity wall, including the luminal edge of the cavity wall, approximately 80% of the pathways were upregulated, and were proinflammatory. The high negative correlation (*r* < −0.6) between pathway expression and *Mtb* burden in the cavity wall was encountered with macrophages, neutrophils, TREM-1, and PKCθ, whereas the high positive correlation (*r* > 0.6) was neuroendocrine (*see* Figure E12); the negative correlation means increased expression of the pathway was associated with improved bacterial containment (lower *Mtb* burden) and thus protective. In contrast, at the air–caseum interface, only 7% of the 60 pathways were upregulated, more than 50% were downregulated (including TREM-1–linked pathways, neuroendocrine, macrophage, and neutrophil pathways), and had the highest *Mtb* burden of all, marking this as the location of immune failure. The highest negative correlation between pathway expression and *Mtb* burden at air–caseum interface were macrophages and neutrophils, whereas high positive correlation was with neuroendocrine pathway, suggesting that these specific pathways play the major role in bacterial containment and, thus, host immunity failure.

Modeling and simulations to explore several possible explanations for this immune failure, including location-dependent reduced influx of immune cells or proliferation or cell death rates, revealed that the most likely mechanism was of *Mtb* killing the immune cells via cell bursting, which was maximal in the cavity center. Other possible mechanisms of cell kill, such as cytotoxic agents released by *Mtb*, will be investigated in separate modeling. The pathways identified here may also be used as biomarkers of the failed bacterial containment in the TB cavity. In addition, molecules targeting these networks could potentially offer new targets for therapy and immunomodulation and to limit transmission (37).

In 2009, Anyanful and colleagues reported that a brief exposure of *Caenorhabditis elegans* to toxigenic *Escherichia coli* conditioned the worms to survive subsequent exposure, because of dopamine signaling linked to innate immune responses (38, 39). In the lung, dopamine receptors are known to directly control alveolar cell inflammatory processes, and indirectly via parathyroid hormone and prolactin release (40, 41). Prolactin promotes proinflammatory innate immune responses via NF-κB and IRF-1 (42). We found decreased expression of dopamine receptor signaling and the downstream pathways (parathyroid hormone, prolactin, and IRIF-1) in TB cavity wall. In addition to dopamine, mGluR signaling, mGluR-dependent synaptic long-term depression formation, and calcium signaling were decreased in parallel. Thus, the neuroendocrine response pathways in the TB cavity that we describe expand the system beyond the dopamine pathway (39). Colonic inflammation in a rat model reduced hippocampal mGluR-dependent synaptic long-term depression formation, which was reversed by chronic administration of minocycline, a drug that also has direct anti-TB effect (43, 44). Moreover, Sarm1 expression decreases mGluR-dependent synaptic long-term depression formation: Sarm1 is a negative regulator of Toll-like receptor signaling, TNF-α, and antiviral cytokines production in mice (45–47).

Our modeling and simulations revealed that neuroendocrine system expression had a high positive correlation with neutrophil and macrophage burst sizes and associated cell-specific death from the *Mtb* infection in TB cavities. Moreover, our simulations demonstrate dose-dependent changes to the Th1 system with perturbation in the neuroendocrine system (*see* Figure E13), a major adaptive immune mechanism effecting *Mtb* control. Furthermore, high correlation coefficients between observed neuroendocrine RPKMs and *Mtb* burden, and the dose-dependent results of the *in silico* perturbation exercises, all suggest that these findings are likely biologically meaningful. Pharmacological manipulation of this neuroendocrine system, once better characterized, may offer a novel approach to reverse failed immunity in the TB cavity, limit *Mtb* burden, and reduce transmission.

There were several potential limitations of our study. First, transcriptomic data were normalized to control subjects without TB rather than pericavitory normal-appearing tissue from the same patient (interpatient vs. intrapatient control subjects). However, viable *Mtb* was commonly present in pericavitory normal-appearing tissue, thus it was far from normal and unsuitable for normalization, making this approach biologically unsound. Second, five South African control subjects had evidence of prior TB, which could introduce biasing toward the null. Therefore, we added a pooled sample of five Americans with no history of TB; the transcriptomic signatures in the control subjects were remarkably similar when compared by nationality (*see* online supplement). Third, our findings pertain to chronic MDR-TB (and MDR-TB plus resistance to quinolones and other drugs) that failed therapy, and may not be generalizable to drug-sensitive TB. Finally, biopsies were only performed from a single cavity rather than from multiple cavities, limited immunohistochemical analysis was undertaken, and functional experiments to interrogate the role of neuroendocrine system were not performed. However, our express goal was to characterize the cavity transcriptome, and we now plan to address the discoveries in a more granular manner.

In summary, construction of a spatially compartmentalized transcriptomic map of MDR-TB cavities identified several hitherto unrecognized pathways involved in the host immune response to TB. Further studies are warranted to investigate the functional characteristics of these pathways and their effect on controlling *Mtb* proliferation.

## Supplementary Material

Supplements

Author disclosures
