# Supplementary material for: Spatial Network Mapping of Pulmonary Multidrug-Resistant Tuberculosis Cavities Using RNA Sequencing
Source: Am J Respir Crit Care Med. 2019 Aug 1;200(3):370–80. doi: 10.1164/rccm.201807-1361OC (PMC6680310; doi:10.1164/rccm.201807-1361OC)
Supplement: Supplements [file rccm.201807-1361OC.html]

Spatial Network Mapping of Pulmonary Multidrug-Resistant Tuberculosis Cavities Using RNA Sequencing | American Journal of Respiratory and Critical Care Medicine

- dheda\_data\_supplement.pdf (4 MB)
- dheda\_supplementary\_table\_e2.xlsx (46 KB)
- disclosures.pdf (301 KB)
