# Supplementary material for: Spatial Network Mapping of Pulmonary Multidrug-Resistant Tuberculosis Cavities Using RNA Sequencing
Source: Am J Respir Crit Care Med. 2019 Aug 1;200(3):370–80. doi: 10.1164/rccm.201807-1361OC (PMC6680310; doi:10.1164/rccm.201807-1361OC)
Supplement: Supplements [file rccm.201807-1361OC_dheda_data_supplement.pdf]

Spatial network mapping of pulmonary multidrug-resistant tuberculosis cavities using RNA sequencing

ONLINE DATA SUPPLEMENT

Sample size

The original sample size for patients with TB that could not be cured by chemotherapy who were undergoing lung resection was chosen using the Wald statistic and pharmacokinetic considerations (1). For that purpose we estimated that a sample size of 14 patients was sufficient, at 80% power, to detect drug clearance differences as small as 30%, with between patient differences of 32% CV (1). That study has a dual aim, pharmacokinetic analyses on antibiotic lesion concentrations across TB cavities, and for RNA-Seqs reported herein. We simultaneously calculated the size of the non-TB [controls] patients for the RNA-Seq analysis (2). We made the assumption of non-normally distributed data with potential for over-dispersion. Thereafter, we computed potential effect size, *d*, derived as median differences in normalized RNA-expression between diseased and normal tissues divided by the pooled standard deviation. In our experimental design, 98 tissues samples would be obtained from 14 cases and 10 samples from 10 control samples. With these data the power observed was 70% with an effect size, *d,* of 0.95, while power of 90% was observed to give an effect size of 1.44. A post hoc consideration using the more recent published data on determining sample size for RNA-Seq samples demonstrated that for 14 cases versus 10 controls, the false positive error was at $\alpha \leq 0.001$ (3).

Institutional Review Board

Ethics and institutional review board permission was obtained from the University of Cape Town, Observatory, South Africa, prior to recruitment of the first patient. All patients gave informed written consent.

Dual PET-CT scanning and reading

Patients were prepared, injected and imaged in accordance with the globally accepted standard FDG PET/CT EANM procedure guidelines (4). After a minimum of 6 hours fasting, blood glucose was monitored, after which a dose of approximately 190 mega-becquerels [MBq] of ^{18}F-FDG was administered intravenously by radiologists. After 60 minutes, the patients were positioned supine in the PET-CT scanner [GEMINI TF Big Bore PHILIPS whole-body scanner] and base of skull to mid-thigh images were acquired in 3-dimensional mode, and reconstructed with attenuation correction. Low dose uncontrasted CT was also performed. Averaged ^{18}F-FDG maximum standardized uptake value [SUVmax] was calculated for the regions of interest, which were the pulmonary cavitary lesions. For the regions of interest, various parameters were calculated, namely, the slice counts in the sampled cavity, total counts, standard uptake volume [SUV] mean, SUV median, SUVmax, SUV pseudo peak [the average of the 3 highest SUVmax's within the cavity], cavity PET volume, mediastinal SUVmax/cavity SUVmax, lung SUVmax/cavity SUVmax and liver SUVmax/cavity SUVmax. Each slice was 4mm and the pixel size was 0.40. Measurements of area were in cm^2 and volume in cm^3. Volume was calculated as the area multiplied by the pixel size per ROI. The total volume was the sum of all the volumes calculated in each region of interest.

Measurement of bacterial burden used for model building

Mtb in biopsies was grown in the MGIT, and monitored for TTP. Next, we grew *Mtb* to 7.0 $\log_{10}$ CFU/mL in log-phase growth and as semidormant bacteria in the laboratory and then serially diluted these 10 fold down to 1 $\log_{10}$ CFU/mL and then simultaneously

inoculated them into the MGIT for TTP and on Middlebrook 7H10 agar for CFU/mL (5). We used these findings to identify the following relationship:

Bacterial burden ($\log_{10}$ CFU/mL)= $8.732*e^{-0.092*TTP}$ -0.085; r^2=0.995

which we used to translate the TTP results from lung biopsies to CFU/mL.

RNA extraction and Next Generation Sequencing

Total RNA from each of the lung tissue samples was extracted inside the BSL3 facility using an miRNeasy mini kit with the Qiazol cell lysis reagent [Qiagen, Maryland, USA]. Briefly, the tissue samples were homogenized in a sterile plastic sample collection bag followed by addition of 1mL Qiazol reagent. Samples were immediately transferred to 2 mL screw capped tubes with addition of 200µL glass beads and bead beaten for 2 min at full speed and followed by incubation at room temperature for 10 min before addition of 200 µL chloroform. RNA purification and elution was done following manufactures' instructions, including on column DNAse-treatment [Qiagen Inc.]. RNA quality and purity were assessed by NanoDrop2000 and Agilent Bioanalyzer RNA 6000 Nano Kit [Agilent Technologies], before library preparation.

Epicenter ScriptSeq Complete Gold Kit Epidemiology was used for the RNA-sequencing library preparation. The two kits we used were - SCL24EP for low input [100-500 ng] and BEP1224 for high input [500ng-2.5 ug] RNA samples. Briefly, samples with RIN number ≥3 were first treated with Ribo Zero solution to remove ribosomal RNA followed by fragmentation using the solution provided in the kit. Next, cDNA was synthesized, purified and a unique barcode was added to each sample by PCR method. Samples were

amplified using 12-14 PCR cycles. Barcoded final libraries were then purified by AMPure XP beads, and the concentration measured using picogreen, and quality assessed by Bioanalyzer. Finally, four samples were pooled onto SE50 [Single End 50bp] Hiseq lane and sequencing was performed on Hiseq 2500 illumina platform. We used ~7 pM of each library for sequencing to obtain a cluster density between 600-800 K clusters/mm2. Cluster generation was performed in an Illumina cBot instrument using TruSeqTM SR Cluster Kits v3, following the manufacturer's protocols [Illumina]. Multiplexed single-read runs were carried out with a total of 57 cycles per run [including 7 cycles for the index sequences], which yielded between 30-40 million sequencing reads per sample.

Illumina HCS 2.2.68.12/RTA 1.18.66.3/SAV 18.46.0 Software was used for real-time image processing and base calling. Raw reads were processed to remove adapter artifacts and to demultiplex the set of reads into its four constituent samples. Reads with no identifiable barcode or with a barcode containing one or more ambiguous base calls were excluded. In the next steps we analyzed the RNA-Seq data for quality control of the reads, and then alignment of reads to human [hg19] and *Mtb* reference genomes [NC_000962] was performed. CLC Genomic workbench [v7] was used for alignment of sequencing reads and statistical analysis of the resulting data to confirm the findings. The steady state expression of various genes was calculated in terms of RPKM values.

Quality control and re-alignment

Sequences were analyzed with FastQC v0.11.3 for quality control analysis. Reads were aligned to human genome version GRCh37 patch 13 using TopHat v2.0.14 at

http://www.ncbi.nlm.nih.gov/projects/genome/assembly/grc/human/. TopHat alignments were sorted and indexed using Samtools v1.2 before the reads were counted using Bedtools multicov v2.23.0. We removed non-RefSeq annotations and annotations without an HGNC symbol. Differential expression analysis was run using DESeq2. An analysis of all sputum samples from lung and non-lung resected patients revealed that they all clustered closely, with no single DEG. We found that 4 of 5 of controls had evidence of prior TB [confirmed by history], and therefore we added a pooled sample of 5 individuals with no history of TB from the USA BioChain Institute [Newark, CA]), as extra controls. We found only 10 DEGs between the Cape Town controls and the pooled control samples, which were mostly mitochondrial origin, consistent with maternal ancestry of the Cape Town population. Therefore, all controls samples from the 10 individuals without TB could be combined. In total, RNA from 69 samples was analyzed.

Statistical and bioinformatics analyses

Differential expression in each of the lesion sites was defined relative to the reads of the same genes in the 10 non-TB controls. Differentially expressed genes (DEGs) were defined as significant if >2-log changes and had a Benjamini-Hochberg adjusted p<0.01. Next, DEGs were imported into Ingenuity Pathway Analysis. We interrogated the whole transcriptome in an agnostic and unbiased fashion by identifying physiological processes most extensively changed in cavitary TB based on the whole transcriptome, including pathways hitherto unrelated to TB. Different numbers of the DEGs that were most highly expressed were examined.

Modular analysis

First, modular analyses were performed as previously described using modules most specific for their annotation, which we developed and validated in the past (6-10). We utilized reads per kilobase per million mapped reads [RPKM] from all the constituent genes in each module. The IL17-inducible gene module has not been previously published and was obtained from transcriptomes of stimulated primary human keratinocytes (9, 10). Significant transcriptional responses to IL17 in comparison to unstimulated keratinocytes were identified with a fold change of at least two-fold (paired t-test $p < 0.05$). Genes that were additionally up-regulated at least two-fold by IFN-γ, TNF-α or IL22 were excluded, and the remaining genes formed the IL17-inducible gene module. This approach reliably ranks the accuracy of gene expression and deconvolutes the data to decipher specific cell type abundance and cytokine or immune stimulus specific modules in tissue samples from transcriptional profiles (6-10).

Immunohistochemistry

After processing in 10% formalin, a selected area of the sampled cavity wall and bronchus connected to the cavity, were scanned and subsequently analyzed in 8/14 patients. The regions were selected and marked so that the area occupied by the various components [encompassing the luminal region, histiocytic band and surrounding chronic inflammation and fibrosis] could be measured digitally within the 200 micron samples. Furthermore, immuno-histochemical staining was conducted for CD4+, CD8+, CD25+, FOXP3+, chromogranin, neurofibrils, s100 protein [neural marker] and CD68+ [macrophage marker]. Briefly, all sections were fixed to glass slides by heating at 60°C

for at least 15 minutes. The procedure was then conducted using the automated IHC/ISH slide staining system, the Ventana Benchmark XT platform [Ventana Medical Systems Inc., Arizona]. The reagents for the Ventana XT are as follows, namely, reaction buffer [concentrate diluted 1:10 prior to use], EZ concentrate [diluted 1:10 prior to use], Liquid Cover Slip [LCS] solution [used neat], CC1 solution [used neat], and SCC solution [used at a dilution of 1:10]. The primary antibodies used are shown in Methods Table 1 below.

Online Methods Table 1. Antibodies used in the immunohistochemical staining procedure

Monoclonal antibody	Clone	Dilution	Catalog number	Supplier
CD4	IF6	1:50	LS-LCD4	Lyka labs
CD25	Tu69	1:100	NCL-CD25-305	Lyka labs
FoxP3	259D/C7	1:150	560044	BD Biosciences
Chromogranin	DAK-A3	1:300	M-0869	DAKO
S100 protein		1:3000	Z-0311	DAKO
Neurofibrils	2F11	1:50	M-0762	DAKO
CD68	KP1	1:1000	M-0814	DAKO
CD8	4B11	1:50	NCL-CD8-4B11	Leica Microsystems

Tonsil lymphoma tissue [provided by anatomical pathology, National Health Laboratory Service] was positively stained with the respective monoclonal antibodies and served as a positive control. Unstained tonsil tissue was used as a negative control. Immuno-imaging was conducted at the Virtual Microscopy Pathology Learning Centre [UCT Health Sciences Campus, Observatory, Cape Town] on an automated advanced imaging solutions system, the Olympus VS120-Z420 [Life Science] at 40X magnification and visualized on the HP ZR2440. Analysis was conducted using ImmunoRatio software [a publicly available web application for quantitative image analysis: http://153.1.200.58:8080/immunoratio/] to assess the percentage area occupied by the

stains. The ImmunoRatio results were thus from the 200 micron selected areas of the cavity wall and bronchus sections, which measured 1.6 mm^3.

Mathematical model

Modeling assumptions

We used DEGs from the RNA-Seq to construct spatial module expression profiles for (i) [T_H1, T_H2, IL10, macrophages, neutrophils] immune responses, (ii) the complement system, and (iii) the signaling factors [TREM-1, neuroendocrine, PKCθ]. The genes that define these immune modules were validated as described in the modular analysis above; those for the signaling factors were as defined in Ingenuity pathway analysis [IPA]. We defined the NEM as the summation of the following IPA defined signaling pathways: dopamine-, glutamate-, synaptic long-term depression, neuronal NOS, and prolactin-signaling. The median RPKM expression values were used to describe the expression patterns of each. Our models assume that the RPKM indicates the level of RNA transcription at the respective locations. High RPKM values are interpreted to represent either high cell densities or pathway expression at the respective locations.

Mathematical models

We developed mathematical models to explain the spatial cell/module expressions at different sites of the cavity, shown in cartoon in online methods Figure 1 below. Next, we developed a simple model that explains the spatial change [from position 1 to 7] in the patterns of each immune response module and the *Mtb* bacterial burden at the different sites of the cavity. We assumed a sequential mapping model, equation 1.

Online Methods Figure 1. Mathematical mapping of expression map on topology/cavity structure

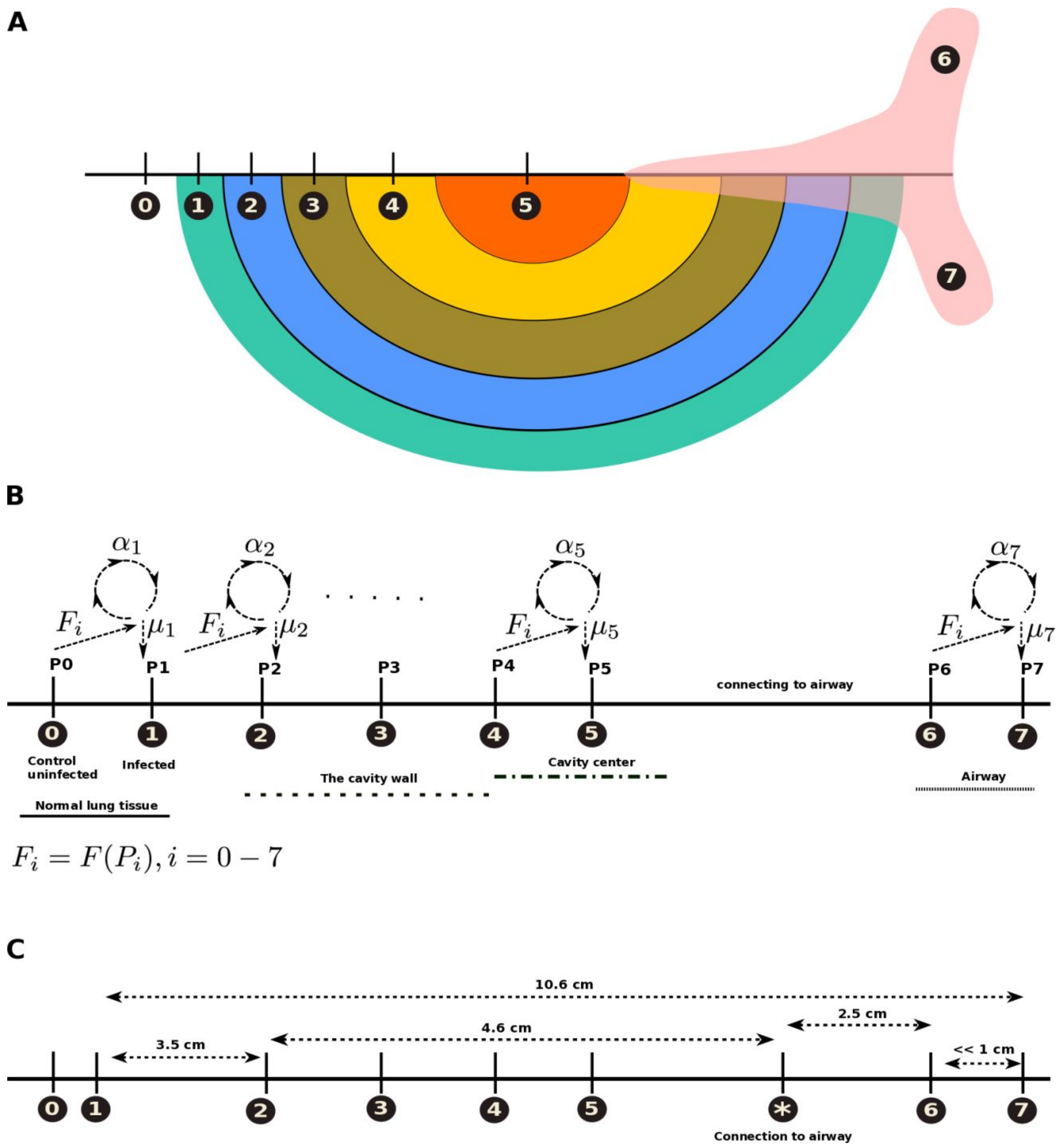

A. Spatial positions counting from position 0, which is theoretical, and represent the expression in lung tissue from non-TB controls. **B.** The parameters α_n and μ_n are location/position *n* specific cell or effector expansion and decay/mortality rates, respectively. **C.** The positions are then replaced by actual ditance in centimeters from normal-appearing tissue; the distances shown are an average; each patient had their own specific distances based on cavity size. The total space mapped is a sphere of about 624 cm^3 of lung tissue and cavity, close to a liter volume.

$$P_n = P_{n-1} + \alpha_n \frac{P_{n-1}}{P_{n-1} + K} - \mu_n P_{n-1}. \quad [1]$$

where $n = 1 - 7$, are the positions on the cavity. P_n is the expression level (or cell population) at each location and P_0 is the expression level in the normal uninfected tissue. The parameters α_n and μ_n are spatial parameters that define position specific cell expansion and decay/mortality rates in figure above**.**

Second, a model [ordinary differential equation] that assumes an increase in expression levels (P) with distance (s) toward the cavity center is used [equation 2].

$$\frac{dP}{ds} = \alpha \frac{P}{P + K} - \mu P. \quad [2]$$

which can be written as a discrete equation

$$P(s + \Delta s) = P(s) + [\alpha \frac{P(s)}{P(s) + K} - \mu P(s)] \Delta s$$

or in a more general form

$$P_{s+1} = P_s + \left[P_s'\right] ds.$$

This model assumes that cell mortality and expansion, or decay of expression is equal at all the locations. If the location specific rates are used, the model will be similar to the first model.

Location specific suppression/decay/mortality

To discriminate cells that are more susceptible to cavity obliteration, we modified the second model to explain patterns at different locations of the cavity. In the TB cavity, based on [1] the PET-CT scans (which depend on uptake of ^{18}F-FDG by activated leuckocytes), [2] histology, [3] the modular analyses, and [4] the heat map of pathways,

we made the assumption that there is either increased cell mortality, or expression decay, or both at the cavity center; in other words *a dynamical sink*.

The model represented by equation (2) can be modified as equation (3) to explain the cavity center increased cell mortality or RNA decay shown in methods figure 2 below.

Online Methods Figure 2. Dynamical sink depiction

$$\frac{dP(s)}{ds} = \alpha \frac{P(s)}{P(s) + K} - (\mu + C_v(s))P(s). \qquad (3)$$

Location dependent mortality (sink)

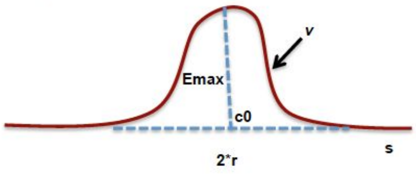

The model is depicted together with the differential equation describing it, showing the cavity potential (E_{max}), the center of the potential well (C_0) which coincides with air-caseum interface at cavity center, and the radius of of the sink (*r*) in centimeters. The parameter α_n is an expansion rate for cell population of effector or *Mtb* burden, while and μ_n is decay/mortality rates for the same.

Modeling the spatial evolution of the coupled modules/cells

The third and final model explains how the different immune cells and effector modules interact together with *Mtb* at the respective positions, and describes the evolution of their corresponding expression levels across the different positions of the cavity. The model is depicted in a cartoon in online methods Figure 3. The model tracks simultaneous cell and effector module interactions and how their respective expression kinetics change in

space, where time is assumed to be invariant and space is a continuum. At each location, we assume an influx of naïve T cells that will commit to either T_H1 or T_H2 cells. The T_H1 and the T_H2 responses may be antagonistic. The adaptive response is stimulated by antigen, the presence *Mtb* bacteria (B) and by antigen presenting cells [macrophages, M].

Online Methods Figure 3. Coupled interactions model

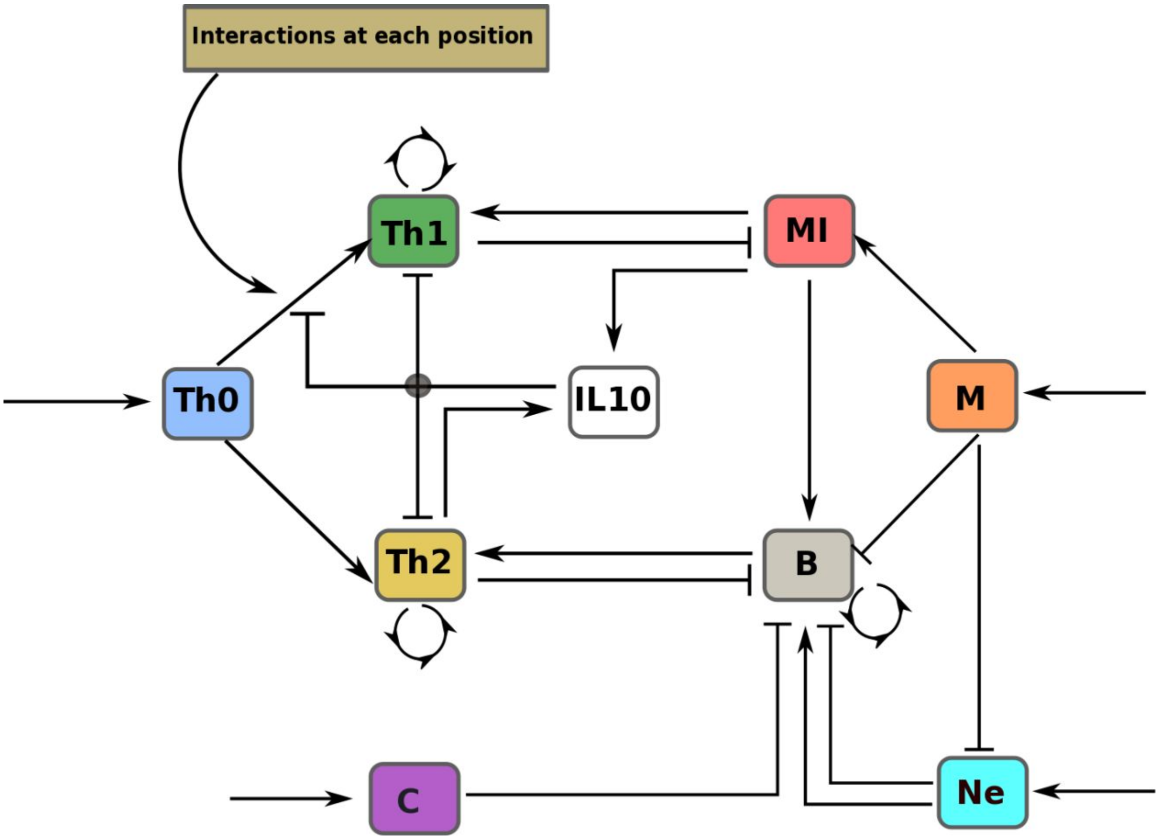

Shown are coupled interactions of different effector systems, cell types and bacteria. After influx, naïve T cells get polarized to be either T_H1 or T_H2 cells. They respond to the presence *Mycobacterium tuberculosis* [bacteria or B], and by antigen presenting cells (macrophages, M), as identified in online methods **Figure 1**. The bacteria also infect macrophages (M) and neutrophils (Ne), at various cavity positions as noted in our H&E stains. Bacteria (B) are presented as either being killed or not, in the equations. Recruitment of macrophages to the site of infection will increase with *Mtb* burden. In certain positions, such as air-caseum fluid phase, activated compliment (C) RNA-seq signal was prominent. Extra-modules for PKCθ, TREM-1 and the neuroendocrine system were added modularly to the coupled interactions shown here. These interactions were modulated by spatial position based on the dynamical sink model described and depicted in online methods Figures 1 and 2 above.

Recruitment of macrophages at the site of infection will increase with *Mtb* burden. We assume that macrophages will induce the secretion of IL10, which may skew the T_H0 selection along the T_H2 pathway. The coupled interactions are shown in cartoon form, where classically activated pro-inflammatory M1 macrophages, neutrophils (Ne), and compliment (C) are shown, and bacteria (B) are presented as either being killed or not.

The model assumes that both neutrophils and macrophages get infected, and contribute to *Mtb* population growth when they burst. In vivo laboratory studies have shown that during the chronic infection phase [10 weeks post infection] recruited macrophages constitute 60% of *Mtb* primary host cells in the lung, while 30% of *Mtb* is in myeloid dendritic cells, and the remaining 10% is in alveolar macrophages (11). When the Mtb grow to a threshold of 20-40 bacilli per cell [termed burst size] they cause necrotic cell death or bursting, which releases bacilli to infect more cells (11). Similarly, neutrophils undergo necrosis when *Mtb* burden reaches a particular size (11-14). However, neutrophils are more susceptible to getting infected than macrophages; also buttressed by H&E stains that showed more *Mtb* infected neutrophils as well as from the literature (14). Also, macrophages are professional phagocytes, and therefore are efficient killers of internalized bacteria. To keep the model relatively simple, we represent both activated and un-activated macrophages by one cell variable, M. Thus the M shown in cartoon is comprised of percentages of M0, M1 and M2 [i.e, is a summation], while cell kill will be effected by activated macrophages but non-activated macrophages will have zero kill.

The model, incorporates other physiologic signaling components [e.g., complement system, TREM-1, PKCθ, and neuroendocrine module] based on DEGs and the heat map in **Figure 2**. The complement system and the neuroendocrine module are assumed to get stimulated by sustained bacterial burden and by cavitation. In the model, we consider the complement system to have anti-microbial effect either via direct kill of the bacteria or else via enhancement of *Mtb* killing by the T_H2 effectors through opsonization, based on published studies (15-17). The role of the neuroendocrine system can switch from enhancing the inflammatory responses in acute infections to suppressing the immune response in a chronic infection, based on dopamine signaling in *C. elegans* studies of fungal and Gram-negative bacillary infection (18-22). Since, our model describes events in a cavity, which is a picture of an unresolved *Mtb* infection in the lung tissue, we assume, the neuroendocrine system suppresses pro-inflammatory responses. Nevertheless, we examined for both assumptions for anti-inflammatory and pro-inflammatory responses in the model, and chose the one based on the data and observed correlations.

In the model, PKCθ is assumed to activate T cell functions. PKCθ is required for T-cell activation, which it achieves via translocation to the immunological synapse in antigen-stimulated T cells to form signaling complexes. This leads to activation of NFκB, activation protein-1 [AP1], and nuclear factor of T cells [NFAT]. From a pathway standpoint, PKCθ upregulation is essential for T cell activation, proliferation, cytokine production, and induction of effective T_H2 responses, as well as by downregulation of regulatory T cell function (23-25). On the other hand, TREM-1 signaling is known to be

pro-inflammatory and to enhance the T_H1 expression and suppress T_H2 expansion, and is highly expressed in inflammatory lesions of infections caused by several bacteria (26, 27). This model does not track the absolute measured number of cells at each location and uses the measured PRKM expression levels as a predictor for the respective cell densities. Therefore, the interaction of the immune cells with the *Mtb* pathogen in the cavity tissue is explained by the following system of equations:

Innate Immune responses

$$\frac{dM_\phi}{ds} = \sigma_m + \gamma_m T_m (B + M_I)/(1 + b_0 E_d) - k_i M_\phi B - (\mu_m + u_{sk}) M_\phi \quad [4]$$

$$\frac{dM_I}{ds} = k_i M_\phi B - k_l M_I Th_1 - k_b M_I - (\mu_I + u_{sk}) M_I \quad [5]$$

$$\frac{dN_e}{ds} = e_N + \gamma_e T_m B/(1 + b_0 E_d) - \gamma_i N_e B - (\mu_n + u_{sk}) N_e \quad [6]$$

$$\frac{dN_I}{ds} = \gamma_i N_e B - \gamma_k M N_I - \gamma_n N_I - (\mu_{nI} + u_{sk}) N_I \quad [7]$$

$$\frac{dC}{ds} = O_c + \alpha B - \mu_c C \quad [8]$$

Adaptive immune responses

$$\frac{dTh_0}{ds} = \delta_0 + \gamma_0 (B + M_I) T_m - \delta_m M_I Th_0 P_\theta - \delta_B B Th_0 P_\theta - (\mu_0 + u_{sk}) Th_0 \quad [9]$$

$$\frac{dTh_1}{ds} = \theta_m \delta_m M_I Th_0 P_\theta/(1 + a_1 IL_{10}) - (\mu_1 + u_{sk}) Th_1 \quad [10]$$

$$\frac{dTh_2}{ds} = \theta_B \delta_B B Th_0 P_\theta/(1 + a_2 Th_1 T_m) - (\mu_2 + u_{sk}) Th_2 \quad [11]$$

$$\frac{dIL_{10}}{ds} = L_0 \delta_L (M_I + Th_2)/(1 + a_3 Th_1) - (\mu_L + u_{sk}) IL_{10} \quad [12]$$

Cell signaling immune factors

$$\frac{dT_m}{ds} = \sigma_T (M + N_e) - (\mu_T + u_{sk}) T_m \quad [13]$$

$$\frac{dP_\theta}{ds} = \sigma_\theta M - \mu_\theta P_\theta \mathrm{g} \quad [14]$$

$$\frac{dE_d}{ds} = \sigma_E B - \mu_E E_d \quad [15]$$

Bacteria

$$\frac{dB}{ds} = N_m k_b M_I + rB + N_n \gamma_n N_e - k_i M_\phi B - k_m B(M_\phi + a_k N_e) - k_c B Th_2(1 + c_k C) - k_g C B - \mu_B B \quad [16]$$

where each symbol describes a model variable shown in online methods Table 2 below:

Online Methods Table 2. Symbols and model variables used in equations

Variable	Biological description
M_ϕ	Macrophages (resting and activated)
M_I	Infected macrophages
N_e	Neutrophils
N_I	Infected neutrophils
C	The complement immune effectors
Th_0	Naïve T cells
Th_1	Differentiated T_H1 cells
Th_2	Differentiated T_H2 cells
IL_{10}	Cytokine interleukin-10
T_m	TREM-1 cell signaling
P_θ	PKC-θ T lymphocytes signaling and activation
E_d	Neuroendocrine secretion and signaling
B	Mycobacterium tuberculosis bacteria (*Mtb*)

The description and values of model parameters are shown in table below.

Online Methods Table 3. Description and values of model parameters

Parameter	Description	Units	Baseline value [range]	Ref
**Infection parameters**				
k_i	Macrophage infection by *Mtb*	Cells/cm	0.012(0-1)	[-]
γ_i	Neutrophil infection by *Mtb*	Cells/cm	0.1(0-1)	[-]
k_b	Macrophage burst rate	Cells/cm	0.07(0-1)	[-]
γ_n	Neutrophil burst rate	Cells/cm	0.08(0-1)	[-]
N_m	Macrophage burst size	Cells/cm	100(1-200)	[-]
N_n	Neutrophil burst size	Cells/cm	100(1-200)	[-]
α_r	*Mtb* replication rate	Cells/cm	1.75(0-2)	[-]
**Effector function**				
k_m	*Mtb* killing by the innate response	Cells/cm	0.01(0-1)	[-]
k_c	*Mtb* killing by the T_H2 effectors	Cells/cm	0.01(0-1)	[-]
c_k	Complement enhancement of T_H2 killing	Scalar	0.001(0-1)	[-]

k_g	MTB killing by complement effectors	Cells/cm	0.01(0-1)	[-]
k_l	Infected macrophage killing by T_H1	Cells/cm	1.4E-5(0-1)	[-]
γ_k	Infected neutrophil killing by macrophages	Cells/cm	0.04(0-1)	[-]
**Stimulation and recruitment**				
γ_m	Macrophage induced recruitment	Cells/cm	E-3 (0-1)	[-]
γ_e	Neutrophils induced recruitment	Cells/cm	E-5 (0-1)	[-]
α	Complement induced production	cm^-1	0.05(0-1)	[-]
σ_m	Macrophage baseline supply	Cells/cm	10 (1-10)	[-]
e_n	Neutrophils baseline supply	Cells/cm	10 (1-10)	[-]
O_c	Complement baseline production	Cm^-1	2 (1-10)	[-]
δ_0	Naïve T cell baseline supply	Cells/cm	0.1 (0-1)	[-]
γ_o	Induced precursor T cell production	Cells/cm	0.1 (0-1)	[-]
δ_m	T_H1 differentiation	Cells/cm	0.01(0-1)	[-]
δ_B	T_H2 differentiation	Cells/cm	E-6 (0-1)	[-]
δ_L	IL10 stimulation	Cm^-1	0.01(0-1)	[-]
θ_m	T_H1 clonal expansion	Scalar	5(1-10^4)	[-]
θ_B	T_H2 clonal expansion	Scalar	5(1-10^4)	[-]
L_θ	IL10 production scale factor	Scalar	5(1-10^2)	[-]
σ_T	TREM-1 secretion/production	Cm^-1	15(0-10^2)	[-]
σ_θ	PKCθ secretion/production	Cm^-1	0.2(0-10^2)	[-]
σ_E	Neuroendocrine secretion/production	Cm^-1	0.005(0-10^2)	[-]
**Inhibition factors**				
a_0	Phagocytes inhibition neuroendocrine	Scalar	1(0-1)	[-]
a_1	T_H1 inhibition by IL10	Scalar	0.01(0-1)	[-]
a_2	T_H2 inhibition by Th1 and TREM-1	Scalar	0.01(0-1)	[-]
a_3	IL10 inhibition by Th1	Scalar	0.01(0-1)	[-]
**Mortality/Decay**				
μ_B	*Mtb* decay/mortality rate	Cells/cm	8.5E-4(0-1)	[-]
μ_m	Macrophage mortality rate	Cells/cm	0.02(0-1)	[-]
μ_I	Infected macrophage mortality rate	Cells/cm	0.01(0-1)	[-]
μ_n	Neutrophils mortality rate	Cells/cm	0.1(0-1)	[-]
μ_{nI}	Infected neutrophils mortality rate	Cells/cm	0.01(0-1)	[-]
μ_c	Complement decay rate	Cm^-1	0.1(0-1)	[-]
μ_0	T_H0 decay/mortality rate	Cells/cm	0.01(0-1)	[-]
μ_1	T_H1 decay/mortality rate	Cells/cm	0.01(0-1)	[-]
μ_2	T_H2 decay/mortality rate	Cells/cm	0.01(0-1)	[-]
μ_L	IL10 decay rate	Cm^-1	0.01(0-1)	[-]
μ_T	TREM-1 decay rate	Cm^-1	0.1(0-1)	[-]
μ_θ	PKCθ decay rate	Cm^-1	0.3(0-1)	[-]
μ_E	Neuroendocrine decay rate	Cm^-1	0.5(0-1)	[-]
**Physical cavity parameters**				
r	Cavity radius	cm	1(0-5)	Cal
E_{max}	Cavity potential energy	Scalar	3.5(0-10)	Est
c_o	Cavity center	Position	Position 5	Cal
ν	Steepness of the cavity potential wall	Scalar	2(0-10)	Est

Parameter Estimation

The models were fitted to the RPKM expression data to estimate parameters that explain the different immune responses across the cavity. Model fitting was carried out in R using the FME package (28). Bayesian statistics with the Markov Chain Monte Carlo [MCMC] methods was used to estimate model parameters for the models assuming non-informative uniform priors and a Gaussian likelihood. Parameters estimated were evaluated using the median values of the MCMC chain of the parameter posterior distributions. Uncertainty in parameter estimates was given by 95% credible intervals calculated from the 2.5% and 97.5% quantiles of the MCMC chain. The MCMC parameter posteriors were drawn of 100,000 runs of the chain with a burnin length of 5,000 runs. MCMC chain convergence was assessed visually and by using chain convergence diagnostic tools in the R Coda packages [ftp://cran.r-project.org/pub/R/web/packages/coda/coda.pdf].

Model parameter sensitivity analysis

We carried out multivariate model parameter sensitivity analysis to assess the influence of parameter variation to model output variability using the Latin Hyper Sampling method. The influence of each model parameter to model output variables [for instance T_H1 cells/module] was quantified with partial rank correlation coefficients [PRCCs] evaluated at a p-value of 0.05. The PRCCs take values between 1 and -1, where a negative value indicates that increasing the magnitude/value of a given parameter will reduce the magnitude of the corresponding model output variable. The magnitude of the

PRCC value indicates the strength of the positive or negative association between the parameter and the model output variable. We used this method (i) to explain how different immune effectors, infection mechanisms, signaling factors control and influence *Mtb* across the cavity. (ii) To show how the different simulated immune responses (effector cells [T_H1,T_H2], monocytes [macrophages, neutrophils], IL10, the complement system, and signaling factors [TREM-1, PKCθ, and the neuroendocrine]) correlate with the bacterial burden in the cavity wall, cavity center and in the airways, respectively.

Model simulations

We used model-fitting to data to completely parameterize the model so that the model could faithfully reproduce the observed patterns in the data. The parameterized model was then used to carry out simulations to explain the underlying biological processes that are beneath the biological observations.

i. The effect of neuroendocrine stimulation in the regulation of immune response

The parameter (σ_E) that models the stimulation of the neuroendocrine signaling was increased using a $\log_{10}$ step size to evaluate how this translate to the change in the expression of the immune modules and *Mtb* burden.

ii. The effect of PKCθ signaling in the stimulation of adaptive immune response

PKCθ signaling of the T_H1 and T_H2 responses was investigated by reducing its corresponding stimulation parameter (σ_θ) using a $\log_{10}$ step size. The effect this has on

bacterial burden across the cavity tissue and how the rest of the innate cells, the complement system, IL10 and cell signaling factors respond, were assessed.

iii. Predicting and explaining underlying biological processes that sustain infection

We computationally tracked and enumerated factors that (i) generate bacteria in the model [that is bacteria extracellular replication, and bursting of infected macrophages and neutrophils], (ii) effector mechanisms that kill bacteria or remove bacteria from the lung tissue [internalization of bacteria by macrophages and neutrophils, successful killing by macrophages and neutrophils, bacteria killing by the complement system, killing by T_H2 effectors]. We then make a comparison to differentiate the factors that are (i) sustaining bacteria populations in the different regions of the cavity [in the wall, at the center and in the airway] and (ii) identify the most effective anti-microbial effectors at the different locations of the cavity.

*iv. Spatial differential influx of effector*s

We also tracked and made a quantitative comparison of the abundances of different immune effectors generated at each location of the cavity. We compared the influx of T_H1 and T_H2 effectors, IL10 stimulation, generation of macrophages and neutrophils and the complement effectors. This analysis was carried out to discriminate regions or locales of the cavity where these effectors are present [and active] and absent [and not active] to give an explanation as to why they are present or absent at the different locations.

REFERENCES

1. Dheda K, Lenders L, Magombedze G, Srivastava S, Raj P, Arning E, Ashcraft P, Bottiglieri T, Wainwright H, Pennel T, Linegar A, Moodley L, Pasipanodya JG, Sirgel FA, Helden PDv, Wakeland E, Warren RM, Gumbo T. Drug penetration gradients associated with acquired drug resistance in tuberculosis pateints. *Am J Resp Crit Care* 2018.

2. Ching T, Huang S, Garmire LX. Power analysis and sample size estimation for RNA-Seq differential expression. *RNA* 2014; 20: 1684-1696.

3. Yu L, Fernandez S, Brock G. Power analysis for RNA-Seq differential expression studies. *BMC Bioinformatics* 2017; 18: 234.

4. Boellaard R, Delgado-Bolton R, Oyen WJ, Giammarile F, Tatsch K, Eschner W, Verzijlbergen FJ, Barrington SF, Pike LC, Weber WA, Stroobants S, Delbeke D, Donohoe KJ, Holbrook S, Graham MM, Testanera G, Hoekstra OS, Zijlstra J, Visser E, Hoekstra CJ, Pruim J, Willemsen A, Arends B, Kotzerke J, Bockisch A, Beyer T, Chiti A, Krause BJ, European Association of Nuclear M. FDG PET/CT: EANM procedure guidelines for tumour imaging: version 2.0. *Eur J Nucl Med Mol Imaging* 2015; 42: 328-354.

5. Gumbo T, Dona CS, Meek C, Leff R. Pharmacokinetics-pharmacodynamics of pyrazinamide in a novel in vitro model of tuberculosis for sterilizing effect: a paradigm

for faster assessment of new antituberculosis drugs. *Antimicrob Agents Chemother* 2009; 53: 3197-3204.

6. Bell LC, Pollara G, Pascoe M, Tomlinson GS, Lehloenya RJ, Roe J, Meldau R, Miller RF, Ramsay A, Chain BM, Dheda K, Noursadeghi M. In vivo molecular dissection of the effects of HIV-1 in active tuberculosis. *PLoS Pathog* 2016; 12: e1005469.

7. Pollara G, Murray MJ, Heather JM, Byng-Maddick R, Guppy N, Ellis M, Turner CT, Chain BM, Noursadeghi M. Validation of immune cell modules in multicellular transcriptomic data. *PLoS One* 2017; 12: e0169271.

8. Byng-Maddick RT, C.; Pollara, G.; Ellis, M.; Guppy, N.; Bell, L.; Ehrenstein, M.; Noursadeghi, M. TNF bioactivity at the site of an acute cell mediated immune response is preserved in rheumatoid arthritis patients responding to anti-TNF therapy. *Front Immunol* 2017.

9. Zaba LC, Suarez-Farinas M, Fuentes-Duculan J, Nograles KE, Guttman-Yassky E, Cardinale I, Lowes MA, Krueger JG. Effective treatment of psoriasis with etanercept is linked to suppression of IL-17 signaling, not immediate response TNF genes. *J Allergy Clin Immunol* 2009; 124: 1022-1010.

10. Nograles KE, Zaba LC, Guttman-Yassky E, Fuentes-Duculan J, Suarez-Farinas M, Cardinale I, Khatcherian A, Gonzalez J, Pierson KC, White TR, Pensabene C, Coats I, Novitskaya I, Lowes MA, Krueger JG. Th17 cytokines interleukin (IL)-17 and IL-22

modulate distinct inflammatory and keratinocyte-response pathways. *Br J Dermatol* 2008; 159: 1092-1102.

11. Repasy T, Lee J, Marino S, Martinez N, Kirschner DE, Hendricks G, Baker S, Wilson AA, Kotton DN, Kornfeld H. Intracellular bacillary burden reflects a burst size for *Mycobacterium tuberculosis* in vivo. *PLoS Pathog* 2013; 9: e1003190.

12. Repasy T, Martinez N, Lee J, West K, Li W, Kornfeld H. Bacillary replication and macrophage necrosis are determinants of neutrophil recruitment in tuberculosis. *Microbes Infect* 2015; 17: 564-574.

13. Corleis B, Korbel D, Wilson R, Bylund J, Chee R, Schaible UE. Escape of *Mycobacterium tuberculosis* from oxidative killing by neutrophils. *Cell Microbiol* 2012; 14: 1109-1121.

14. Eum SY, Kong JH, Hong MS, Lee YJ, Kim JH, Hwang SH, Cho SN, Via LE, Barry CE, 3rd. Neutrophils are the predominant infected phagocytic cells in the airways of patients with active pulmonary TB. *Chest* 2010; 137: 122-128.

15. Dunkelberger JR, Song WC. Role and mechanism of action of complement in regulating T cell immunity. *Mol Immunol* 2010; 47: 2176-2186.

16. Dunkelberger JR, Song WC. Complement and its role in innate and adaptive immune responses. *Cell Res* 2010; 20: 34-50.

17. Merle NS, Church SE, Fremeaux-Bacchi V, Roumenina LT. Complement System Part I - Molecular mechanisms of activation and regulation. *Front Immunol* 2015; 6: 262.

18. Anyanful A, Dolan-Livengood JM, Lewis T, Sheth S, Dezalia MN, Sherman MA, Kalman LV, Benian GM, Kalman D. Paralysis and killing of *Caenorhabditis elegans* by enteropathogenic *Escherichia coli* requires the bacterial tryptophanase gene. *Mol Microbiol* 2005; 57: 988-1007.

19. Anyanful A, Easley KA, Benian GM, Kalman D. Conditioning protects *C. elegans* from lethal effects of enteropathogenic *E. coli* by activating genes that regulate lifespan and innate immunity. *Cell Host Microbe* 2009; 5: 450-462.

20. Kawli T, Tan MW. Neuroendocrine signals modulate the innate immunity of Caenorhabditis elegans through insulin signaling. *Nat Immunol* 2008; 9: 1415-1424.

21. Kawli T, He F, Tan MW. It takes nerves to fight infections: insights on neuro-immune interactions from C. elegans. *Dis Model Mech* 2010; 3: 721-731.

22. Kawli T, Wu C, Tan MW. Systemic and cell intrinsic roles of Gqalpha signaling in the regulation of innate immunity, oxidative stress, and longevity in *Caenorhabditis elegans*. *Proc Natl Acad Sci USA* 2010; 107: 13788-13793.

23. Rosse C, Linch M, Kermorgant S, Cameron AJ, Boeckeler K, Parker PJ. PKC and the control of localized signal dynamics. *Nat Rev Mol Cell Biol* 2010; 11: 103-112.

24. Zanin-Zhorov A, Ding Y, Kumari S, Attur M, Hippen KL, Brown M, Blazar BR, Abramson SB, Lafaille JJ, Dustin ML. Protein kinase C-theta mediates negative feedback on regulatory T cell function. *Science* 2010; 328: 372-376.

25. Brezar V, Tu WJ, Seddiki N. PKC-Theta in Regulatory and effector T-cell functions. *Front Immunol* 2015; 6: 530.

26. Sharif O, Knapp S. From expression to signaling: roles of TREM-1 and TREM-2 in innate immunity and bacterial infection. *Immunobiology* 2008; 213: 701-713.

27. Colonna M. TREMs in the immune system and beyond. *Nat Rev Immunol* 2003; 3: 445-453.

28. Soetaert K, Pedzoldt,T. Inverse modelling, sensitivity and Monte Carlo analysis in R using FME. *J Stat Sofw* 2010; 33: 1-28.

ONLINE RESULTS TEXT

ONLINE FIGURES

Figure E1. PET-CT scans and readings in patients with MDR-TB

Figure E2. Histology findings on hematoxylin and eosin stains in patients with MDR-TB

Figure E3. Bacterial burden expressed as time to positivity

Figure E4. RNA sequence realignment by chromosome

Figure E5. Principal component analysis of all DEGs from MDR-TB patients and controls

Figure E6. Heat maps of expression of constituent genes in modules

Figure E7. Calcium and neuroendocrine signaling at air-caseum interface

Figure E8. Magnification of neuroendocrine module fit of dynamical sink model

Figure E9. Standard model for relationship to pathway expression by spatial position

Figure E10. Model 2 failed to fit/explain the data

Figure E11. Partial rank correlation coefficients between immune parameters

Figure E12. Relationship between *Mtb* burden and physiologic pathways by position

Figure E13. Neuroendocrine perturbation induced changes in other immune pathways

Figure E14. Immunohistochemistry for selected cell markers and proteins

TABLES

Table E1. Clinical notes on the 14 patients with MDR-TB who underwent surgical therapy

Table E2. Extended dataset for IPA [see attached Excel file]

Table E3. Estimated parameters for the separate modules.

TEXT RESULTS

Mathematical model integrating networks, spatial location, and bacterial burden

Mathematical models could be used to summarize this multipathway and topology constrained structure. We calculated median RPKM values of all genes in each pathway, based on the rationale detailed in the methods, and related these to the cavity position and mycobacterial burden at each cavity position. First, we fitted the standard linear model (model 1) to the data. This standardly used approach demonstrated a poor fit to the data (**Figure E9)**. Therefore, we created a mathematical map of expression levels (or cell populations) at each cavity location (P_n), depicted and detailed in methods section (model 2). We wrote the ordinary differential equation 2 for the model, which assumed increase in expression levels (P) with distance (s) toward the cavity center. This model, which assumes that cell mortality and expansion are equal at all the locations, failed to explain the depletion of immune cells and pathway expression at the center of the cavity as shown in **Figure E10**. Thus there was a mechanism, or a biological process, the model and its assumptions did not capture.

Model simulation-perturbation experiments

What is the relationship between *Mtb* burden and each physiological module expression at each cavity position location (cavity position) in simulations? **Figure E12** shows strong negative correlation between bacterial burden, macrophage and neutrophil number in all cavity positions, while the high negative correlation of *Mtb* burden with of TREM-1

and PKCθ was encountered only in cavity wall. T_H1 and T_H2 effector expression had poor correlation with *Mtb* burden. On the other hand, there was high correlation between neuroendocrine module expression and *Mtb* burden, which was of the same magnitude as between neutrophil and macrophage counts and *Mtb*. Therefore, we examined the effect of perturbing the neuroendocrine system on *Mtb* burden in simulations.

We also performed simulations to probe the changes of Mtb burden, as well as all other systems *in toto* when the putative neuroendocrine system is perturbed to a specified degree [i.e., in a dose-effect fashion], with results shown in **Figure E13.** The figure shows profound neuroendocrine "dose-dependent" changes in bacterial burden, with increase in *Mtb* burden as neuroendocrine signaling increases. **Figure E13** also shows negative relationships of the NEM with expression of T_H1, IL10, complement, and infection of macrophages and neutrophils, which was constrained by spatial position. This gives a first glimpse of the expected system-wide and bacterial burden changes in response to a hypothetical pharmacological or vaccine-targeted perturbation within this type of integrated system.

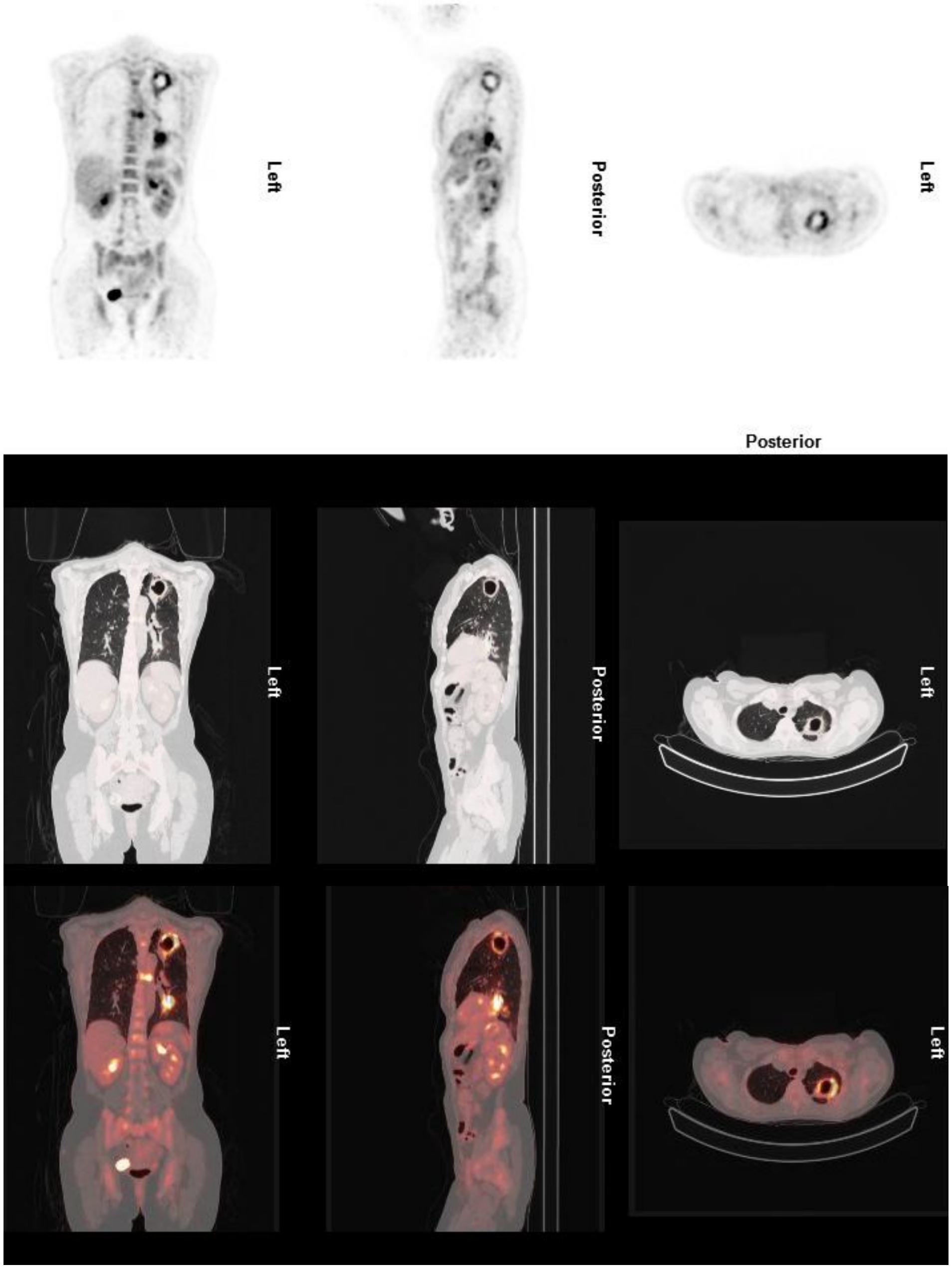

Highest counts in slice in cavity	Total counts in cavity	Highest SUV mean in cavity	Highest SUV Max in cavity	Cavity SUV Pseudo Peak	Highest SUV Median in Cavity	Cavity PET Volume	Cavity/ Mediastinal SUVmax	Cavity/ Lung SUV max	Cavity/Liver SUV max
320.00	2,717.68	2.70	6.07	6.00	2.65	76.54	4.23	14.57	3.82

Figure E1. **PET-CT scans and readings in MDR-TB patients.** An example of scans from a patient with cavitary MDR-TB (patient #11).

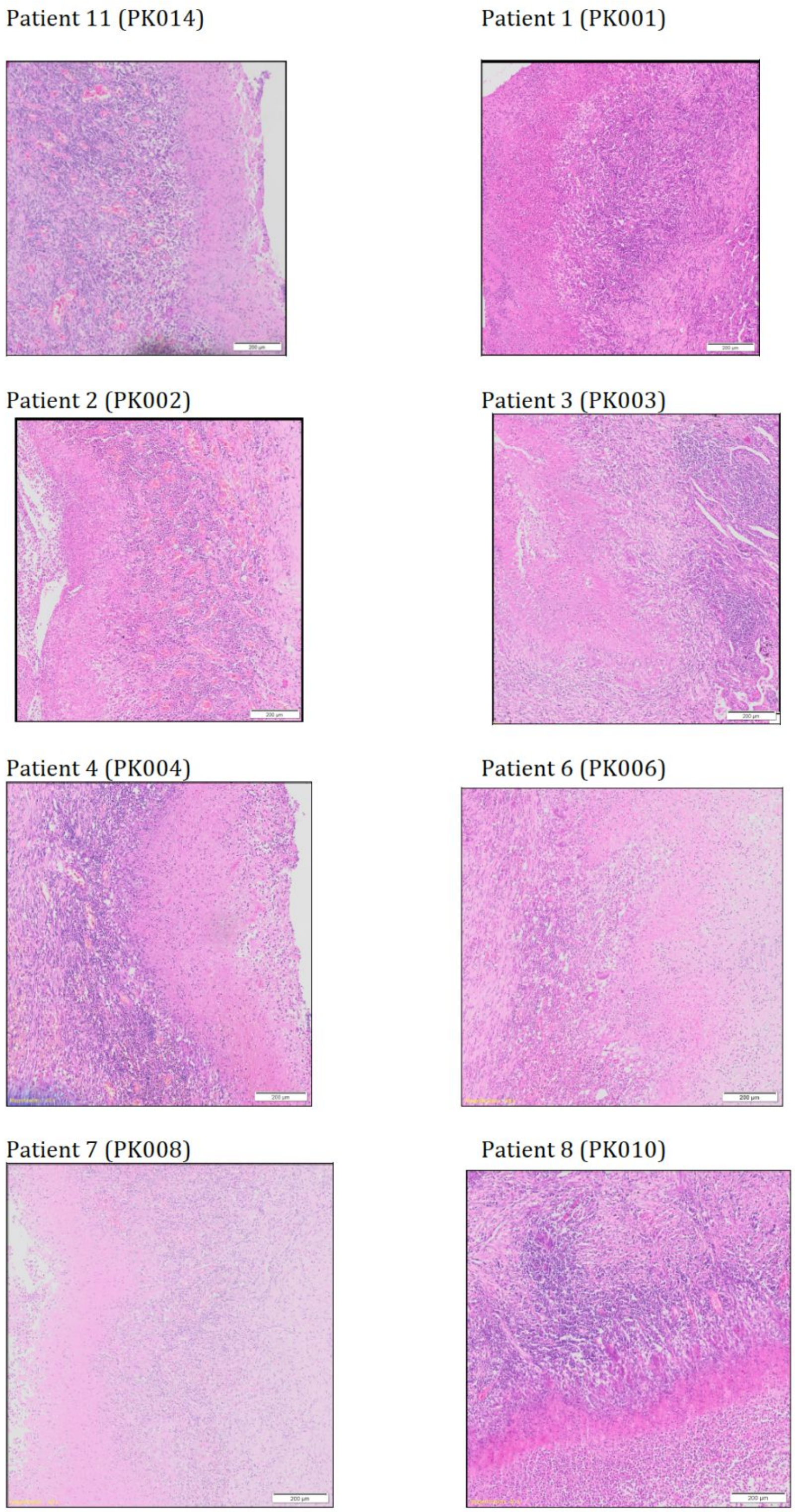

Figure E2. **Histology findings on H&E stains in patients with MDR-TB.** Readings for each are given in a column in **Table E1**. The luminal surface of the cavity wall was

covered by granulomatous inflammation in 9/14 [64%] patients whereas 5/14 [36%] had other types and distribution of epithelia in the same position [3 had respiratory/bronchial epithelium and 2 squamous metaplasia interspaced with granulomatous inflammation].

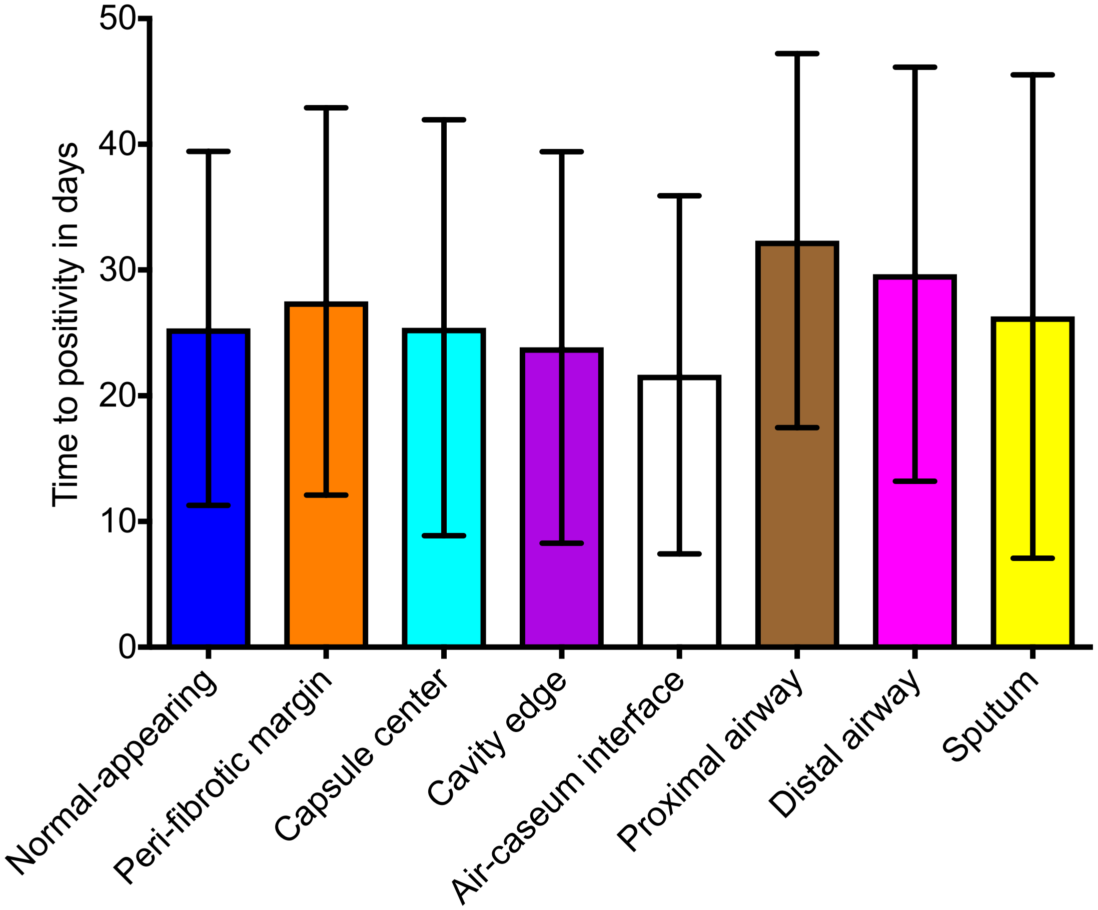

Figure E3. Bacterial burden expressed as time to positivity. Shown are means, with error bars indicating standard deviation. Time-to-positivity [TTP] is inverse to bacterial burden, the higher the TTP the lower the bacterial burden. Thus, the lowest TTP at the air-caseum interface in the cavity center [position 5] indicates the highest mean bacterial burden in colony forming units per gram.

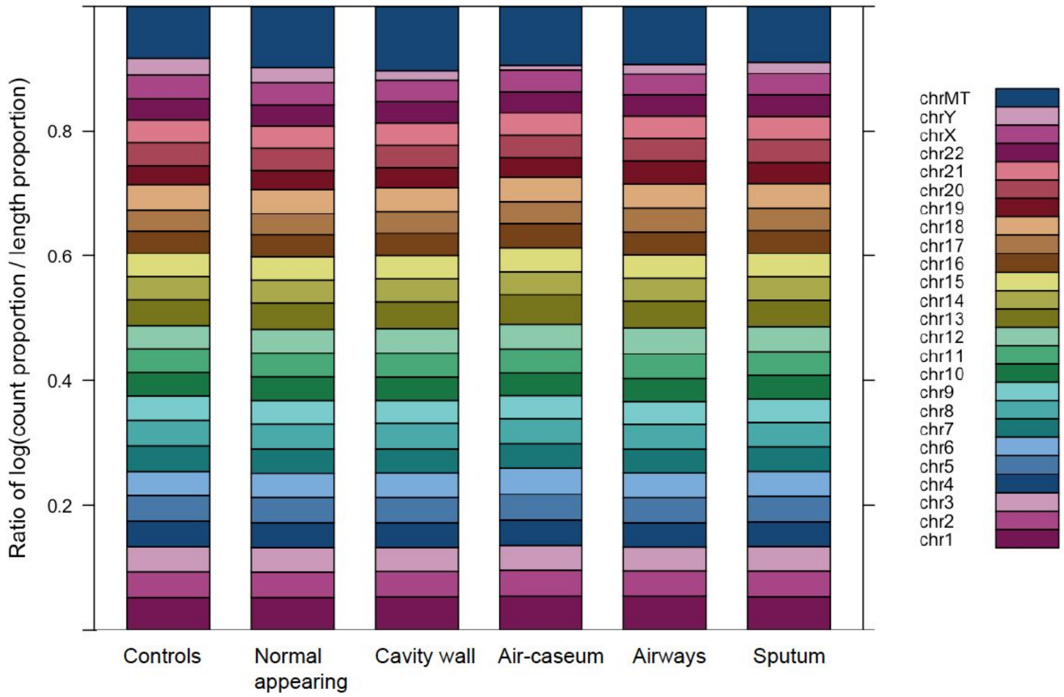

Figure E4. Sequence realignment by chromosome. Realignment of RNA sequence reads to the human genome by specific chromosome; since chromosomes have different sizes the read counts were normalized to the number of genes on a chromosome.

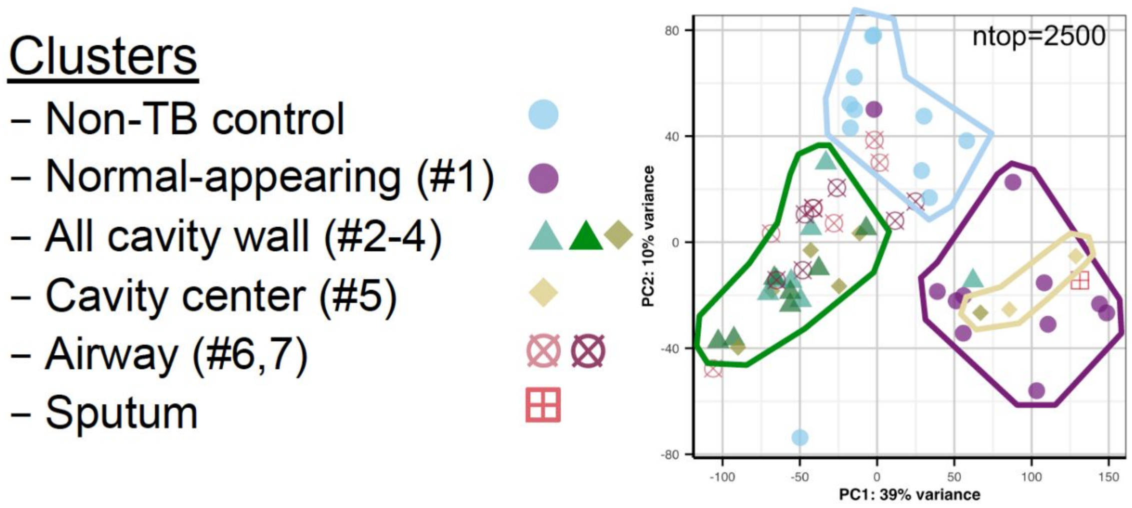

Figure E5. **Principal component analysis of all DEGs from MDR-TB patients and controls**

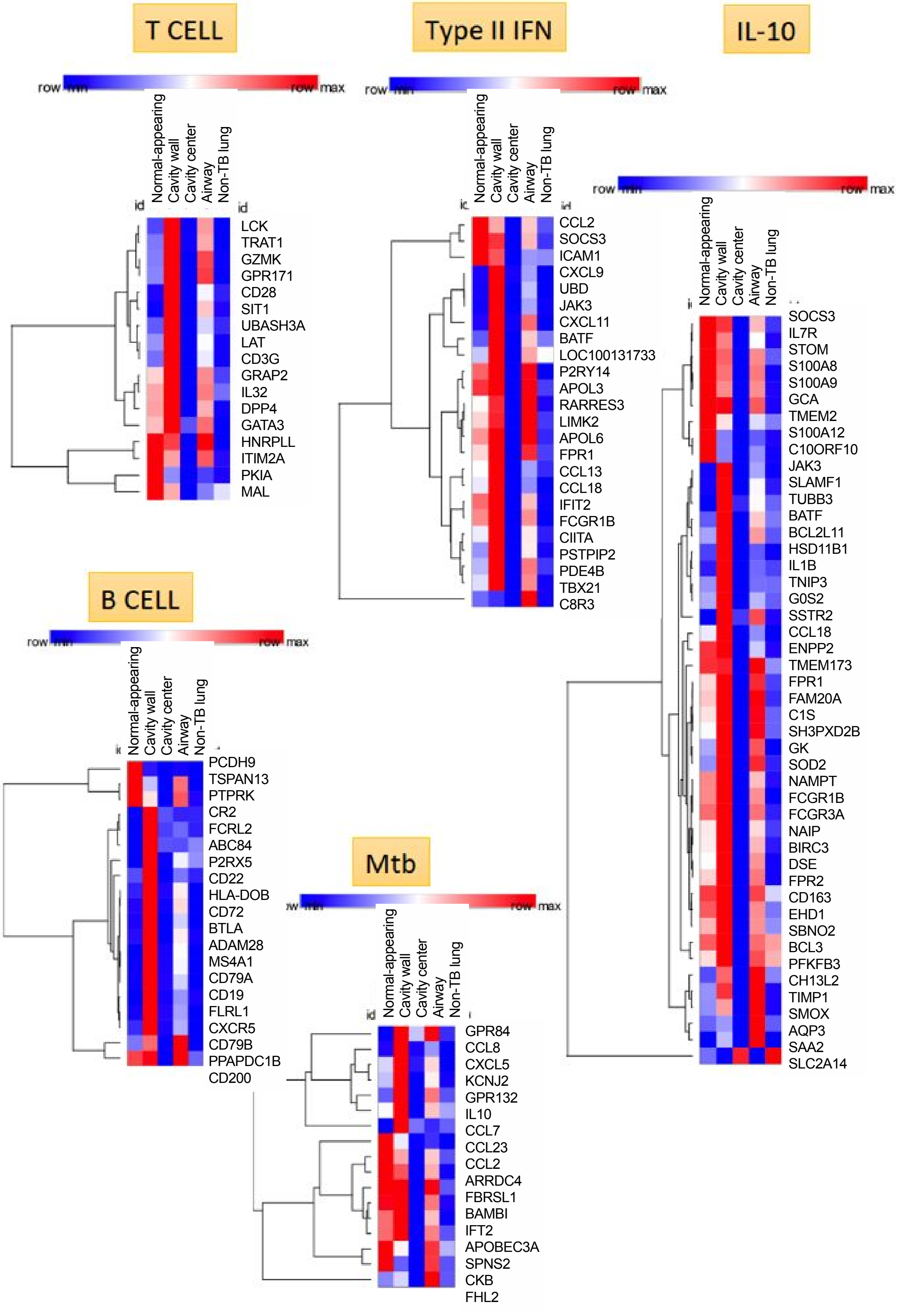

Figure E6. Heat maps of expression of constituent genes in each module

Shown are examples of heat maps for genes in T-cell, B-cell, Type II interferon, IL10 and the *Mtb* modules. The heat maps show that in each module multiple genes were involved; the same picture was encountered in all other modules. The heat maps also show that genes were upregulated in some parts of the cavity such as the wall, but downregulated in some parts such as the center, hence gene expression was spatially constrained. For all modules shown here, gene expression was highest in cavity wall and lowest in cavity center.

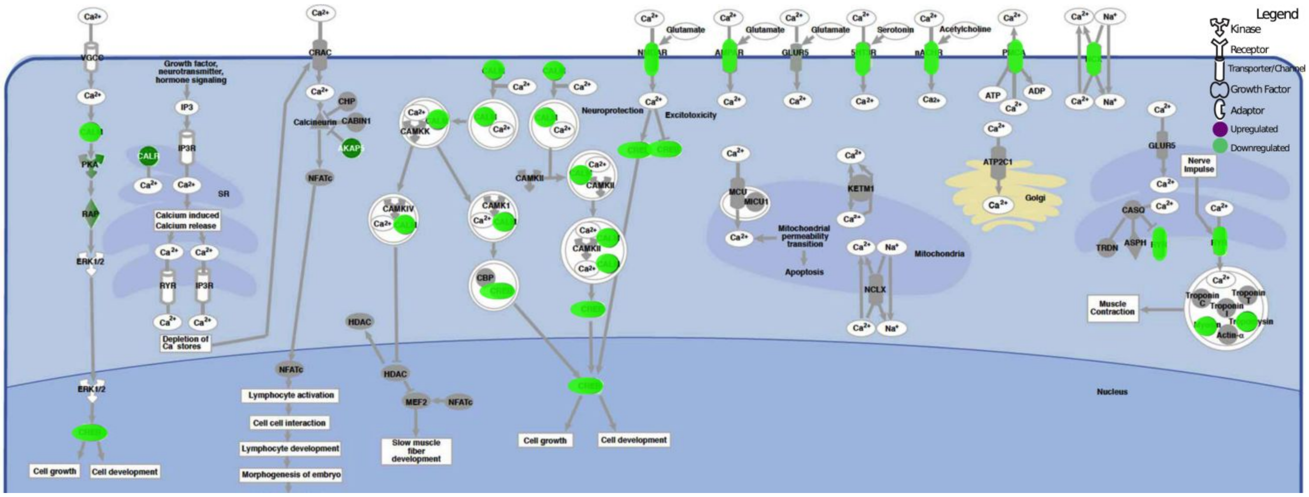

Figure E7. Calcium and neuroendocrine signaling at air-caseum interface.

There was a wholesale decrease in genes encoding Ca^{2+} signaling at the cavity center, in parallel to receptors of neurotransmitters such as NMDAR and AMPAR [ionotropic glutamate receptors], the serotonin receptor 5HT3R and acetylcholine receptor nACHR. Genes for receptor-operated channels were significantly downregulated, as were ryanodine receptors (encoded by *RYR*) which lead to release of Ca^{2+} from the endoplasmic reticulum to cytosol. However, calreticulin (gene *CALR*) which binds and inactivates Ca^{2+} in endoplasmic reticulum to decrease signaling was upregulated. Finally, the two systems associated with Ca^{2+} extrusion from cytosol, the Na Ca^{2+} exchanger (NCX) and plasma membrane Ca^{2+} ATPase (PMCA), are shown with decreased expression.

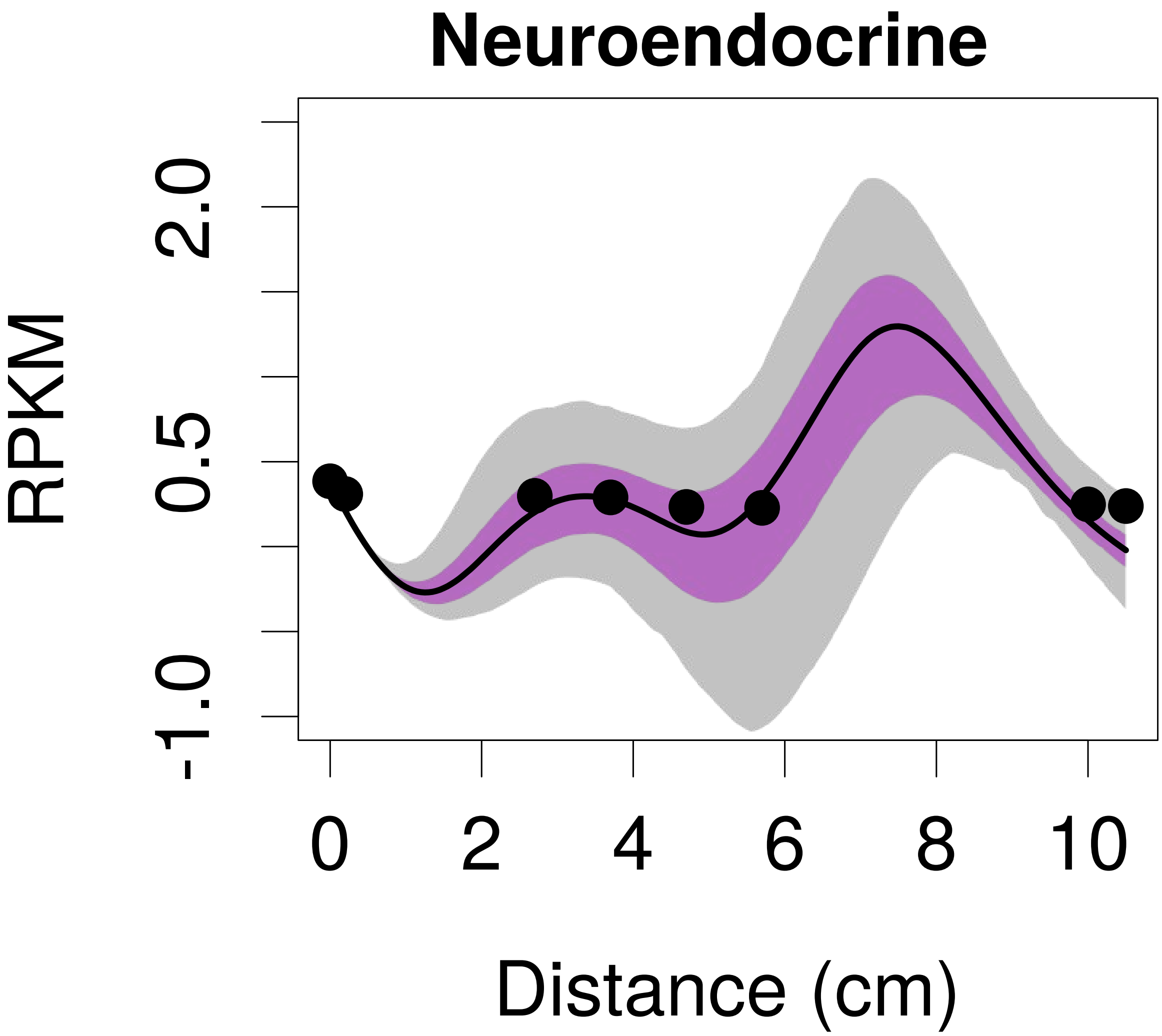

Figure E8. Magnification of neuroendocrine module fit of dynamical sink model

The scale on the neuroendocrine system module if Figure 5 has been changed and the figure magnified to demonstrate the shape of the neuroendocrine dynamical sink model.

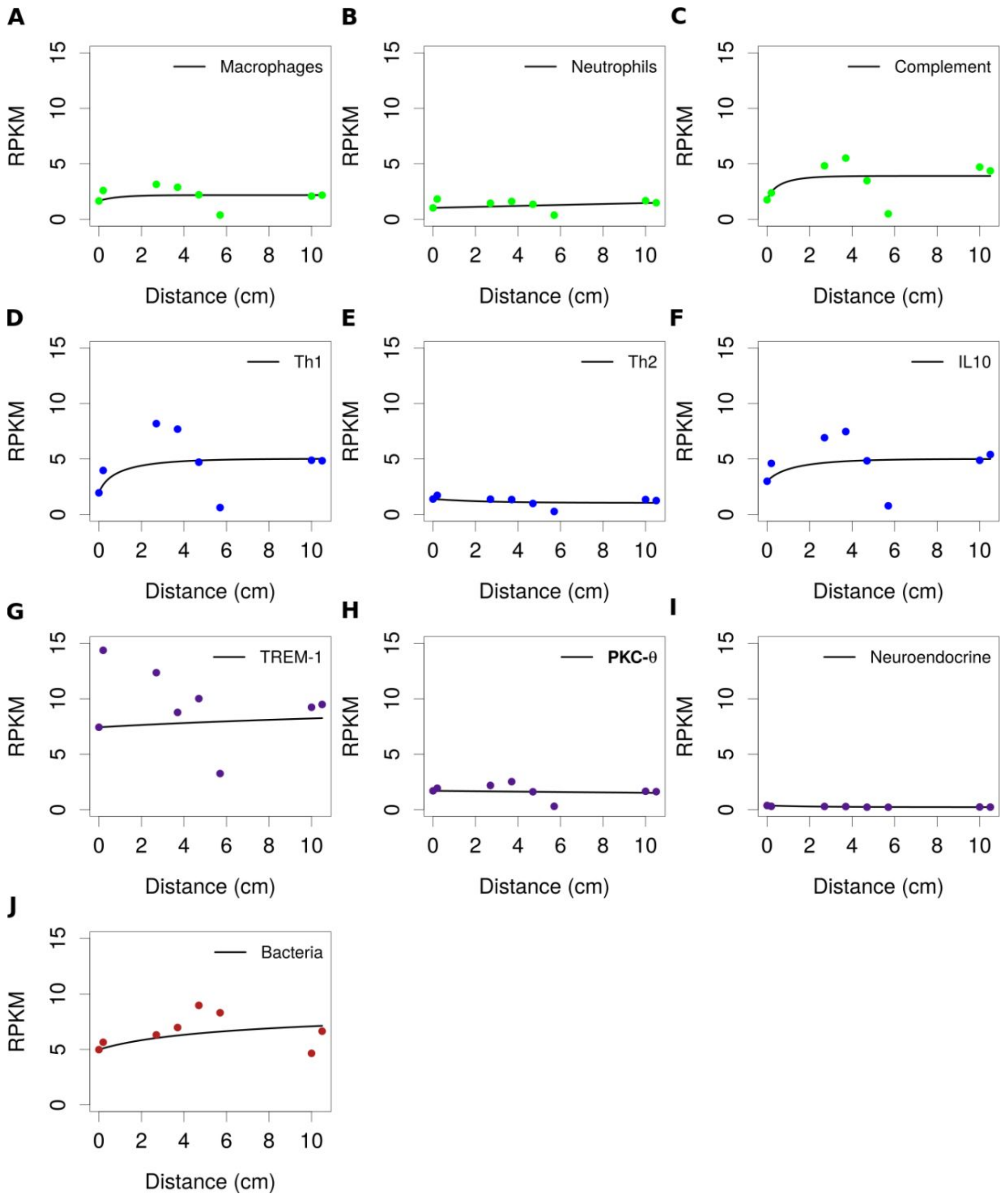

Figure E9. Standard model for relationship to pathway expression by spatial position. The spatial position is given by centimeters from controls (0 cm), through normal-appearing tissue to the cavity center. The model failed to explain the depletion of immune cell expression at the center of the cavity. This means that there is a mechanism or a biological process the model did not capture.

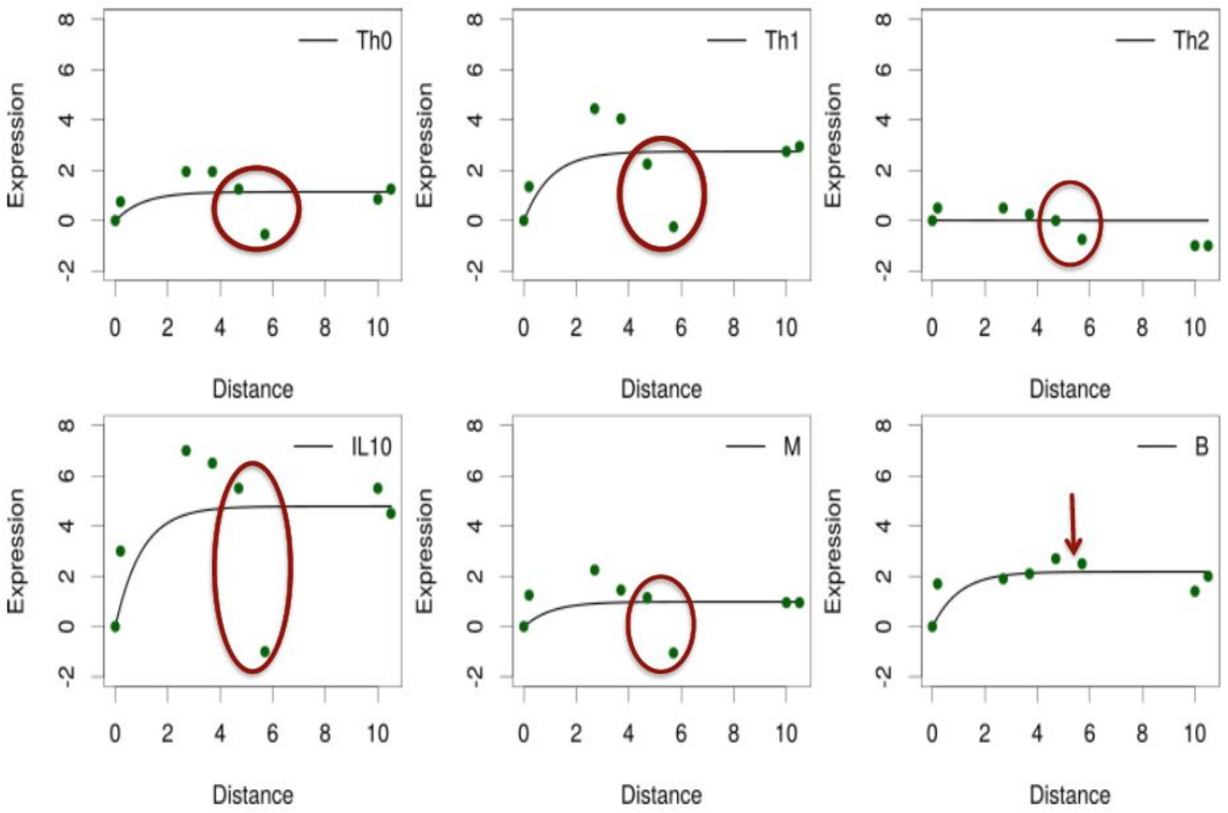

Figure E10. Model 2 failed to fit/explain the data. The model fails to explain the depletion of immune cell expressions at the center of the cavity. This suggests there is a mechanism or a biological process the model did not capture.

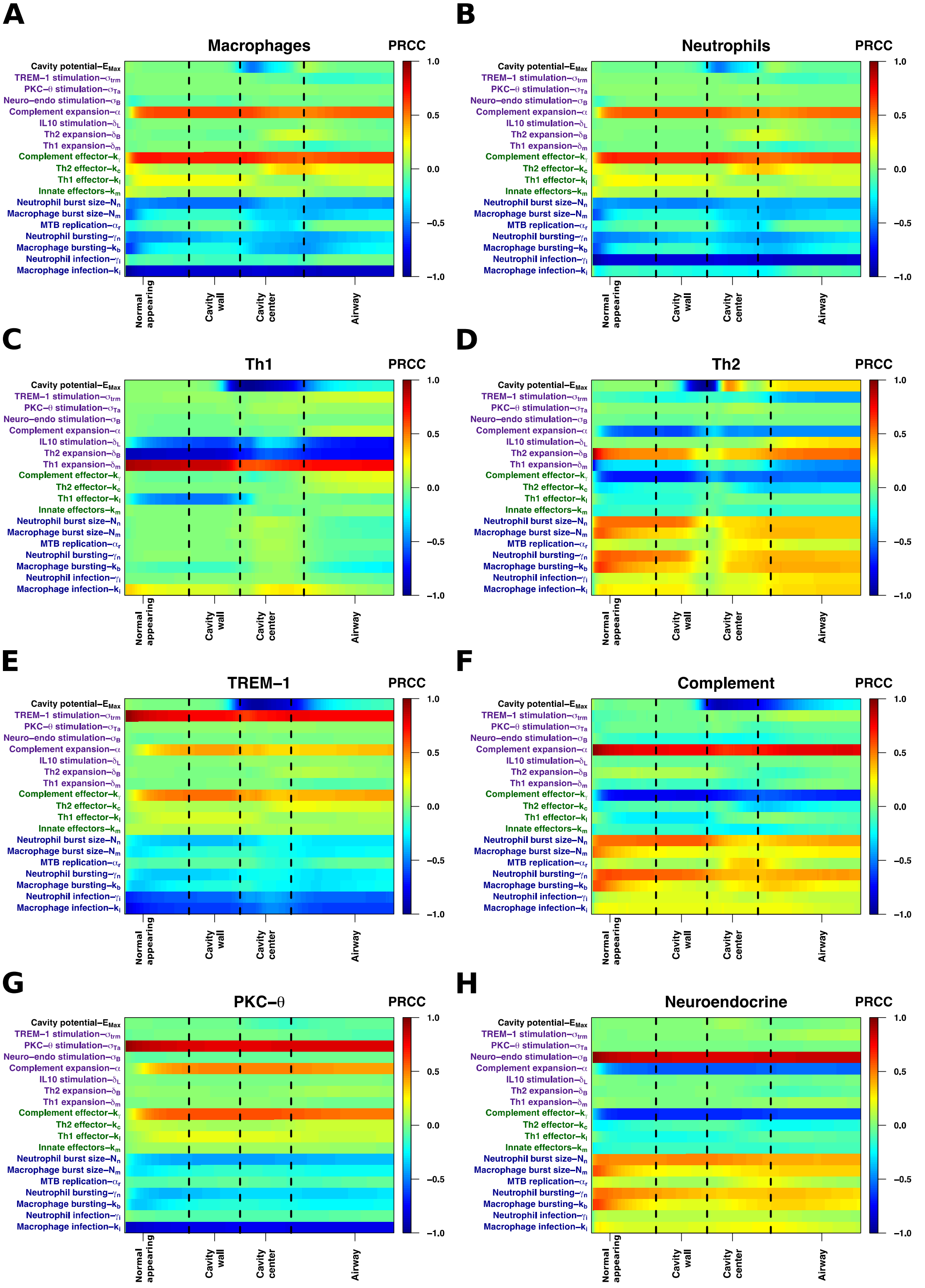

Figure E11. Partial rank correlation coefficients between immune parameters.

Partial rank correlation coefficients (PRCC) are shown on a color scale, with red color

intensity for positive correlation and blue for negative correlation. PRCC is more powerful at determining the sensitivity of a parameter that is strongly monotonic yet highly nonlinear when compared to ordinary Partial Correlation Coefficient analysis. **(A)**. Number of macrophages was negatively correlated with macrophage infection and burst size, but also neutrophil intracellular *Mtb* burden burst size. The most intense positive correlation was for macrophages versus complement expansion, as measured by RPKM values between these two parameters. **(B).** Neutrophils essentially demonstrated similar relationships as shown for macrophages. **(C)**. For the T_H1 system, as expected there is inverse correlation with T_H2. The important finding is negative correlation with cavity potential intensity in the cavity center, consistent with decay of the T_H1 system at that position, as seen with modular analysis. **(D)**. On the other hand, T_H2 showed the highest negative correlation with compliment. **(E).** TREM-1 had the highest negative correlation with neutrophil and macrophage infection. **(F).** Complement had a moderate positive correlation with neutrophil and macrophage bursting, mostly in normal-appearing tissue and cavity wall. **(G)**. PKCθ had the highest positive correlation with complement and the highest negative correlation with macrophage infection. **(H).** The neuroendocrine system had the highest correlation with macrophage and neutrophil bursting in normal-appearing tissue, and high negative correlation with complement.

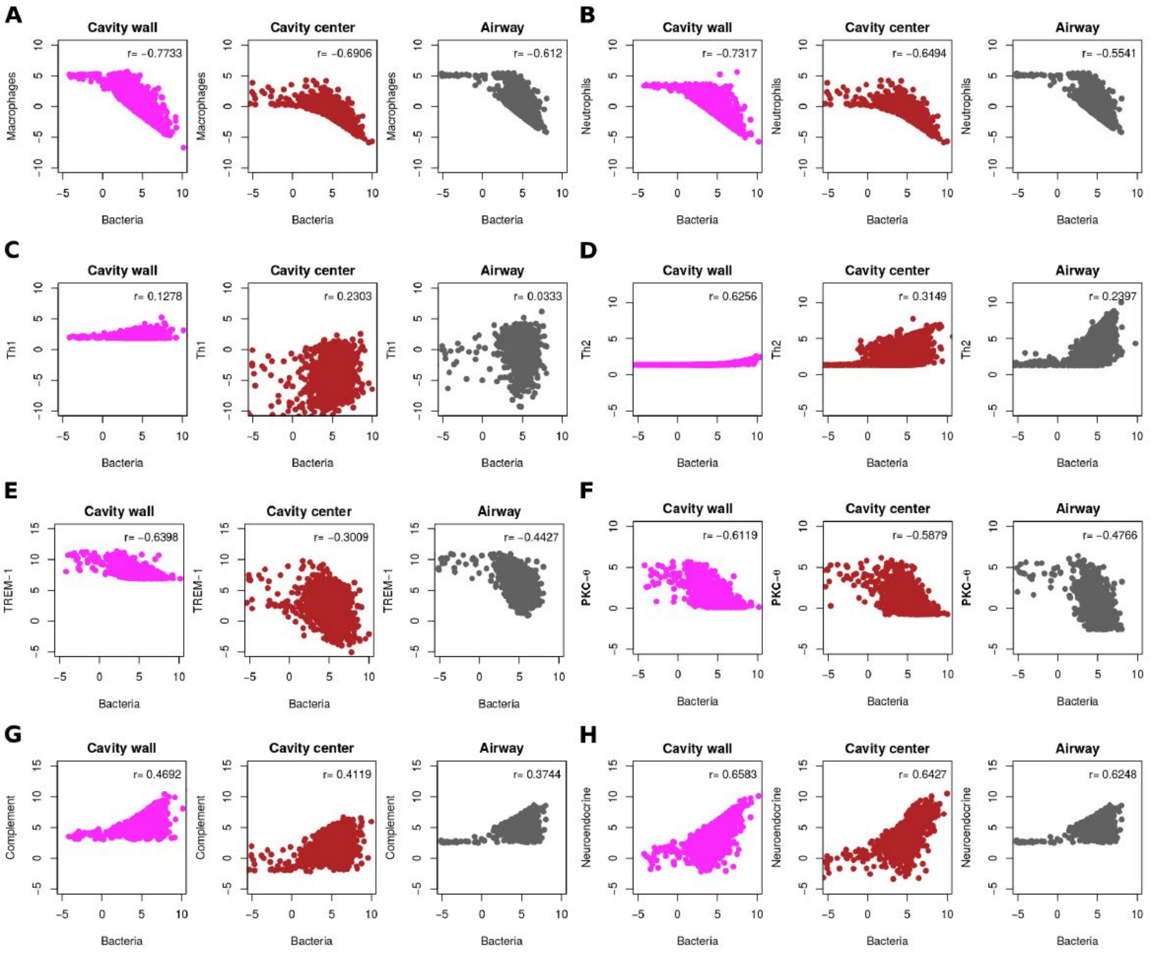

Figure E12. Relationship between *Mtb* burden and physiologic pathways by position. Y-axis shows bacterial burden in $\log_2$ CFU/mL, while X-axis shows $\log_2$ RPKM expression. Partial rank correlation coefficients [PRCCs] r was calculated, with strong correlation defined as an r>0.6 or <-0.6, and poor correlation as an r<0.4 or <-0.4. **(A)** Macrophage numbers and **(B**) neutrophil numbers were associated with a high negative r, thus bacterial burden decreased as number of these cells increased. **C-D** shows poor correlation between T_H1 and T_H2 effector expression and *Mtb* burden, while **E-F** show high negative correlation of TREM-1 and PKCθ and *Mtb* burden in cavity wall. **H**. There was high correlation between neuroendocrine module expression and *Mtb* burden,

which was of the same magnitude as between neutrophil and macrophage counts and *Mtb*.

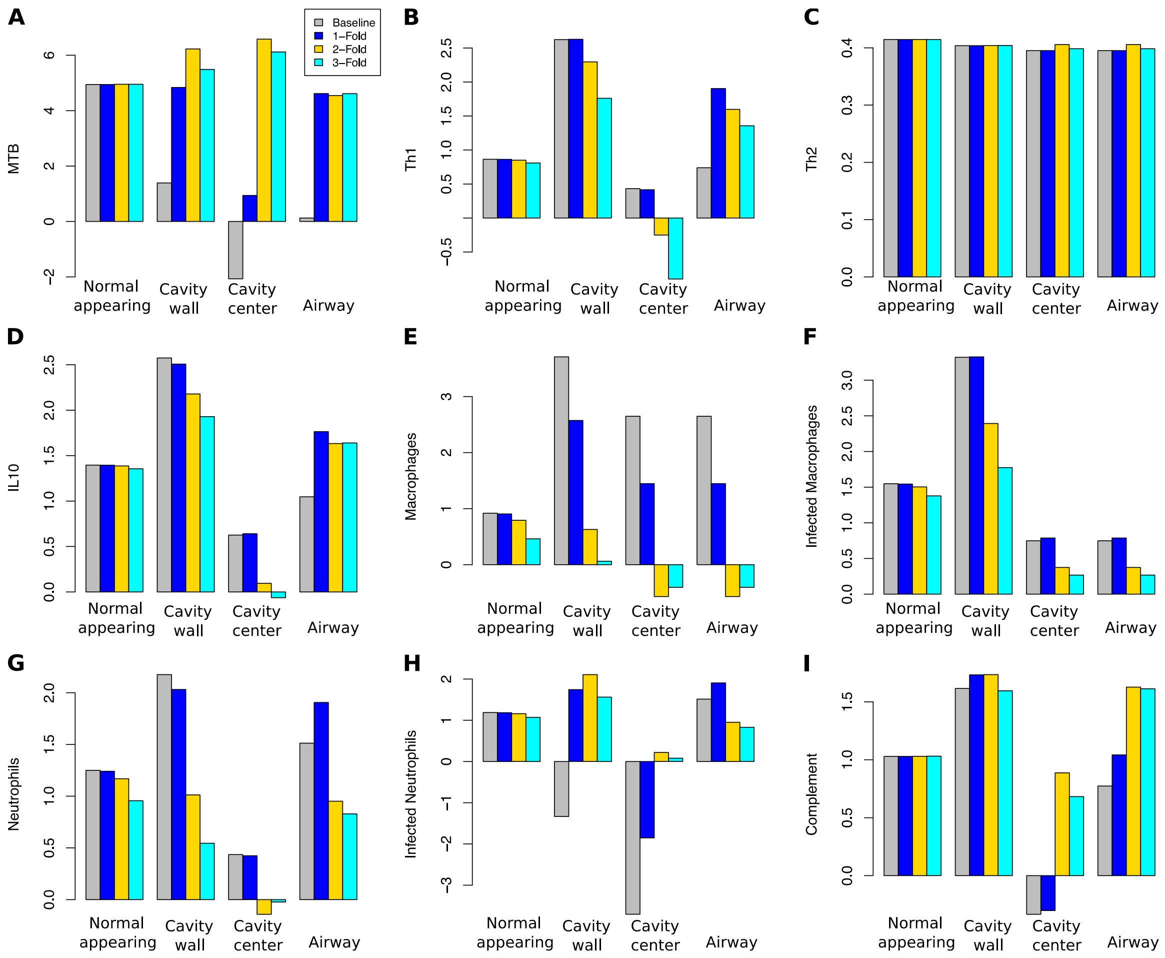

Figure E13. Neuroendocrine perturbation induced changes in other immune pathways. Changes in several immune parameters with changes in neuroendocrine signaling shows a strong "dose-response" effect in the cavity wall and center for T_H1 responses, IL10, and numbers of macrophages and neutrophils as well as infected cells. Relationships are also explored for complement, and shows large change in the cavity center and airway as neuroendocrine signaling was perturbed in the model. The relationship with bacterial [MTB] burden is particularly striking and is encountered in all positions in cavity [with differences by topological position] except in normal-appearing tissue.

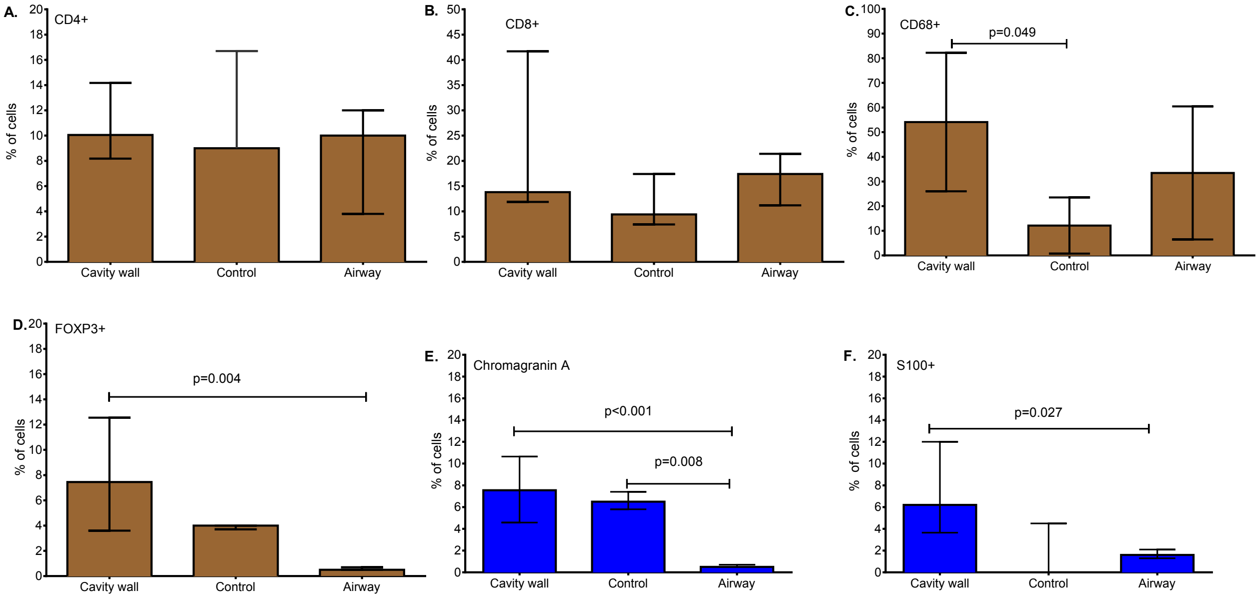

Figure E14. Immunohistochemistry for selected cell markers and proteins. For each of the immunohistochemical stains, we show the median values with inter-quantile ranges of the ImmunoStain ratio. **A.** CD4+ cells demonstrated that the % of these cells were similar between normal lung tissue from patients without TB [control] and cavity wall and airways. **B.** CD8+ cells were also no different compared to controls. **C.** CD68+, expressed on macrophage lineage, was higher in cavity wall compared to controls, and consistent with H&E results. **D.** While the difference in CD25+ cells (up in T cell activation), a median ratio of 18.73 in cavity wall, 15.6 in airway, and 29.1 in control, were not significantly different, FOXP3+ cell values were 7.5 in cavity wall, 0.5 in airway, and 4.0 in controls [Kruskal-Wallis test p=0.004]. This means there are spatial differences in regulatory T cell activity, up in cavity wall but a "hole" in center. **E**. Chromogranin A is the precursor to several peptides that have autocrine and paracrine function. In normal lung tissue, neuroepithelial bundles have the highest abundance in airways compared to normal lung parenchyma; here they were lower in airway compared

to non-infected control lung, which means reduction in airways due to TB. **F**. The polyclonal rabbit anti-S100 that we used reacted strongly with human S100B, a marker of neuroglia, and was higher in the cavity wall.

Table E1. Clinical notes on the 14 patients with MDR-TB who underwent surgical therapy.

Age [Yrs]	Gender	PET cavity volume [mm^3]	Type of surgery	Gross pathology summary	Histopathology summary	TTP (days)
14	F		Wedge resection of superior segment of LUL	40mm apical cavity with a 22mm nodule positioned directly below.	Cavity lined by necrotizing granulomatous inflammation and scarring. Eosinophils were present at the edge of the cavity. ZN stains showed mycobacteria within the cavity lumen and wall.	29.57±10.18
24	F	388.86	Left pneumonectomy	Destroyed left lung contained multiple cavities: 1 large cavity (80 mm in diameter) and 2 smaller 20mm cavities.	Large apical cavity lined by respiratory epithelium and necrotizing granulomatous inflammation. Smaller cavities lined by inflamed granulation tissue and caseating granulomatous inflammation. ZN stain showed numerous AFB throughout cavity, but sparse in the cavity wall.	14±3.46
33	F	35.65	Left upper lobectomy	Left upper lobe contained a bronchus leading to a small cavity measuring approximately 25mm in diameter and a second, larger 50mm cavity.	Cavities lined by necrotizing granulomatous inflammation and keratinizing squamous metaplasia. ZN stain shows AFB in cavity as well as AFB in neutrophilic exudate on the luminal surface of cavities.	21±12.54
29	M		Right pneumonectomy	Two dominant cavities present (1x 15mm and 1x25mm).	Sections of the apical cavity showed an area of necrotizing granulomatous inflammation, with the remainder lined by bronchial epithelium. ZN stain on the outer limits of the cavity showed a single AFB.	34.25±19.5
41	M	49.60	Left pneumonectomy	30mm apical cavity and a second larger 40mm cavity.	Cavities lined by bronchial epithelium and squamous metaplastic epithelium	NEG
23	M	27.14	Wedge resection of superior segment of LUL	20mm diameter subpleural cavity. Numerous fibrocaseous nodules were present throughout the lung.	Sections of the cavity showed marked necrotizing inflammation. AFB were present in the cavity and the surrounding area.	13.29±5.22
26	F	241.02	Right pneumonectomy	Large cavity occupying the right upper lobe measuring 55 x 30 x 40mm, smaller cavity was present in the lower lobe.	Cavities showed caseous granulomatous inflammation of the wall; ZN stain showed AFB in cavity luminal surfaces and surrounding areas.	9.29±4.39
40	F	14.91	Left pneumonectomy	One 50mm diameter cavity present in the superior part of the lower	Sections of the cavity lined by necrotizing granulomatous inflammation in luminal region, ZN stain showed numerous	25±0

				lobe	AFB.	
29	M		Right upper lobectomy	A 50mm subpleural cavity, with a fibrocaseous nodule of 6mm adjacent to the cavity and multiple nodules up to 3mm diameter.	Sections of the cavity wall and luminal surface lined by caseous granulomatous inflammation. Single AFB seen in the wall of the cavity wall, but clusters of AFBs in cavity center.	31.14±12.27
16	F		Left pneumonectomy	One large cavity (40 mm in diameter) was present	Abundant caseous material in the lumen. Palisaded histiocytes on the periphery surrounded by fibrosis. ZN stain revealed only 1-2 AFBs within the luminal surface of the cavity.	NEG
49	F	76.54	Left upper lobectomy	A large cavity (45 mm in diameter was present) and an adjacent single apical nodule.	Cavity lined by caseous granulomatous inflammation. ZN stain revealed AFBs in cavity wall.	39±10.63
50	F		Wedge resection of superior segment of LUL	Large apical cavity 40 mm diameter and an adjacent fibrocaseous nodule of 7mm.	Cavity lined by active necrotizing granulomatous inflammation. ZN stains showed numerous AFB in cavity and luminal surface, and scant AFB in cavity wall.	16.86±4.45
42	F		Right pneumonectomy	40mm cavity in upper lobe and a smaller 20mm diameter cavity in the apex of the lower lobe.	Both cavity walls lined by a layer of caseation and necrotic neutrophils. ZN stains on the cavity lumen, luminal surface, wall and surrounding tissue showed numerous AFB.	NEG
48	F		Left pneumonectomy	An apical cavity (25mm in diameter) and surrounding tubercles were present in the lower lobe, and a second smaller cavity (10mm) was present in the inferior part of the upper lobe.	Both cavities lined by necrotizing granulomatous inflammation. ZN stains showed numerous AFB in lumen, on luminal surface, and in the cavity wall, and in the surrounding area.	9.14±3.85

Table E3: Estimated parameters for the separate modules.

Module/Parameter	T_H1	T_H2	IL-10	Macrophages	Neutrophils	TREM-1	PKC-θ	Complement	Neuroendocrine	*Mtb*
**Model with sink**										
α	14.99	2.65	14.99	10.30	1.02	3.37	7.56	10.69	0.63	14.99
u	0.01	1.0	0.01	0.73	0.13	0.47	0.84	0.13	0.50	0.01
K	0.1	0.1	0.3	5.0	1.9	1.7	3.7	0.1	0.1	0.1
E_{max}	7.97	1.44	7.26	2.58	0.49	1.88	1.9	7.86	0.001	0.39
**Model without sink**										
α	14.99	1.13	14.99	4.55	0.50	14.99	0.24	14.99	0.72	14.99
u	0.46	0.49	0.47	1.0	0.01	0.01	0.03	1.0	0.52	0.05
K	0.01	0.24	0.01	0.01	10.0	0.01	9.8	0.01	0.19	0.01

A table of estimated parameters using a single equation model. α's represent the RNA transcription rates and μ's are the corresponding decay rates. The parameter K represents the half-maximal stimulation for RNA increase/expansion. The model was fitted to the separate immune response modules [i] assuming a region with increased RNA suppression/decay measured using the value of E_{max} [model 3] and [ii] assuming equal RNA decay across all regions of the cavity [model 2].
